# Plasmid replication-associated single-strand-specific methyltransferases

**DOI:** 10.1093/nar/gkaa1163

**Published:** 2020-12-03

**Authors:** Alexey Fomenkov, Zhiyi Sun, Iain A Murray, Cristian Ruse, Colleen McClung, Yoshiharu Yamaichi, Elisabeth A Raleigh, Richard J Roberts

**Affiliations:** New England Biolabs Inc., 240 County Road, Ipswich, MA, USA; New England Biolabs Inc., 240 County Road, Ipswich, MA, USA; New England Biolabs Inc., 240 County Road, Ipswich, MA, USA; New England Biolabs Inc., 240 County Road, Ipswich, MA, USA; New England Biolabs Inc., 240 County Road, Ipswich, MA, USA; Université Paris-Saclay, CEA, CNRS, Institute for Integrative Biology of the Cell (I2BC), Gif-sur-Yvette, France; New England Biolabs Inc., 240 County Road, Ipswich, MA, USA; New England Biolabs Inc., 240 County Road, Ipswich, MA, USA

## Abstract

Analysis of genomic DNA from pathogenic strains of *Burkholderia cenocepacia* J2315 and *Escherichia coli* O104:H4 revealed the presence of two unusual MTase genes. Both are plasmid-borne ORFs, carried by pBCA072 for *B. cenocepacia* J2315 and pESBL for *E. coli* O104:H4. Pacific Biosciences SMRT sequencing was used to investigate DNA methyltransferases M.BceJIII and M.EcoGIX, using artificial constructs. Mating properties of engineered pESBL derivatives were also investigated. Both MTases yield promiscuous m6A modification of single strands, in the context SAY (where S = C or G and Y = C or T). Strikingly, this methylation is asymmetric *in vivo*, detected almost exclusively on one DNA strand, and is incomplete: typically, around 40% of susceptible motifs are modified. Genetic and biochemical studies suggest that enzyme action depends on replication mode: DNA Polymerase I (PolI)-dependent ColE1 and p15A origins support asymmetric modification, while the PolI-independent pSC101 origin does not. An MTase-PolI complex may enable discrimination of PolI-dependent and independent plasmid origins. M.EcoGIX helps to establish pESBL in new hosts by blocking the action of restriction enzymes, in an orientation-dependent fashion. Expression and action appear to occur on the entering single strand in the recipient, early in conjugal transfer, until lagging-strand replication creates the double-stranded form.

## INTRODUCTION

The role of DNA modification in restriction-modification (RM) mechanisms of prokaryotic cells was established >50 years ago (1,2). However, the prevalence of non-RM-associated modification and the diversity of associated functions remains incompletely understood (3). Long-read, modification-sensitive SMRT sequencing technology has facilitated sequencing and assembly of a wide variety of genomes and also clarified the modification repertoire. Detection of the modification status of bases is possible for m6A, m4C and oxidized forms of m5C modified bases (m5hC and 5caC) (4). Recently, high throughput analysis of 230 diverse bacterial and archaeal methylomes strikingly revealed that almost 50% of organisms harbor Type II DNA methyltransferases (MTase) homologs with no apparent cognate restriction enzyme (RE) (5).

These ‘orphan’ MTases sometimes exhibit patterns of incomplete methylation that distinguish them from RM system MTases (6,7), enabling roles in regulation of gene expression and DNA replication in diverse bacterial and archaeal phyla. Two well-studied examples in *Escherichia coli* are the orphan DNA MTases Dam and Dcm, which play roles in mismatch DNA repair, DNA replication and phase variation of protein expression (6). In alpha-proteobacteria, *Caulobacter crescentus* CcrM is also well studied, and evolved independently to regulate numerous cellular functions (e.g. (8,9)). Additional examples are accumulating: M.CsaII modifies 76% of sites in the native host (10), M.EcoGX modifies 34% (11). Selective modification by the conserved CamA MTase is also widespread in the population of *C. difficile* (12).

Genome and methylome analysis of total DNA from two pathogenic strains of *Burkholderia cenocepacia* J2315 and *E. coli* O104:H4 revealed the presence of two related and apparently silent MTases not previously characterized. Both are coded by plasmid-borne ORFs, in pBCG2315 for *B. cenocepacia* J2315 (13,14) and pESBL for *E. coli* O104:H4 (11).

Genetic and biochemical study of these two MTases led us to propose that they modify single-stranded DNA in coordination with DNA Polymerase I *in vivo*. Here we present a short introduction to replicons used for this study and the *in vivo* mechanisms that reveal ssDNA substrates.

### Vegetative plasmid replication modes

Plasmid families have characteristic replication-initiation modules (*ori* regions, also called replicons) (15) with typical copy number per chromosome within the host. Host replication machinery is used for elongation, so the *ori* regions can be thought of as adaptors to a common downstream output.

Plasmids with ColE1 and p15A replication origins (used here) use a PolI-dependent replication initiation mechanism common to many high-copy plasmids of Proteobacteria (16). The host's vegetative RNA polymerase synthesizes an initiator RNA (RNAII) and, from the opposite strand, a copy-number regulator (RNAI). The RNAII:DNA hybrid stably displaces the parental leading strand. The hybrid is processed by RNAse H to provide the primer for DNA extension by PolI. If RNAI anneals to RNAII, processing and primer extension are prevented, lowering the number of plasmid copies. RNAII initiation is regulated by Dam modification of the promoter (17). This replication is resistant to translation-inhibiting drugs, making it effectively independent of cellular mechanisms that coordinate division and replication (18). Plasmids with distinct sequences in the RNAI-RNAII annealing region do not cross-inhibit, and thus are compatible, even though both rely on the same initiation mechanism. ColE1 and p15A are compatible for this reason.

Though replicon classification is incomplete (19), both low-copy pSC101 (used here) and many characterized conjugal plasmids (including the F factor) employ plasmid-encoded initiator proteins. These proteins promote assembly of the DNA Polymerase III replisome during vegetative replication (15). Like the host cell, pSC101 requires only the 5′→3′ Exonuclease activity of PolI, for Okazaki fragment maturation (20) but not the polymerase activity. Prolonged exposure of single-strand regions has not been reported for such replication strategies.

### Conjugal replication in the donor and the recipient

In addition to vegetative replication during cell growth, conjugal plasmids use a distinct replication program to move from one cell to another (21). The DNA that is transferred to the recipient is single-stranded, entering unidirectionally from a unique transfer origin, *oriT* (22,23). Transfer begins with a specific nick at *oriT*, mediated by a nickase (a relaxase component). The nickase forms a covalent attachment to the 5′ side of the nick, leaving the 3′ side of the nick to prime leading strand synthesis in the donor. The displaced strand is transported into the recipient through a complex channel, with the nickase attached.

Second-strand synthesis occurs in the recipient. The entering ssDNA is made duplex initially by DNA Polymerase I. This employs RNA primers synthesized from promoters designated F*rpo* (*ssiD;* in F) or *ssi2* and *ssi3* (in the IncI1 plasmid Col1b-9). These promoters are recognized by the vegetative RNA polymerase *only* when they occur in single strands (24–26). The transcripts serve two functions: as primers for lagging-strand DNA synthesis (24), and as translation templates for establishment functions, which include anti-host-defense functions.

The recipient cell frequently regulates DNA entry with defense strategies, including restriction activities and SOS-regulated suicide mechanisms. The gene neighborhood in the early-transferred conjugal DNA includes many anti-defense factors that thwart these cellular regulators of DNA exchange: ArdA, which inhibits action of Type I RM systems; PsiA and PsiB, which suppress induction of the SOS response (and resident prophages) by entering single strands; ParB homologs, and Ssb proteins, which bind to single strands (27). These functions are always coded for in the same orientation relative to *oriT* ensuring they are transcribed and translated from the single-strand that is transferred into the recipient (28). Expression requires the recipient RNAP (29). M.EcoGIX and its homologs are found here, in the orientation that allows expression from the anti-defense promoters.

Complete establishment for conjugal plasmids requires resumption of ds→ds replication. Willetts and Wilkins (30) point out that at least two recipient priming mechanisms are needed for F' transfer and establishment, one RifR in a wild-type recipient (aka replisome assembly with PolIII) and one RifS in a DnaB-defective host (Clamp helicase, so replisome-deficient). Complete transfer required DnaE (Pol III).

### Phage M13 biology

Single-strand DNA phages develop through ssDNA and dsDNA life phases (31). M13 filamentous virions comprise a circular single-stranded DNA molecule encased in a thin flexible tube made up of protein VIII, with protein III at one end. The F factor pilus is required by pIII as a receptor. Once inside the cell, replication occurs in three phases; establishment (ss→ds), amplification (ds-ds), and secretion (ds→ss). For viral secretion, the phage employs a rolling circle replication mechanism, with virions assembled on the displaced single strand, which are then secreted through the cell envelope. Replication during viral secretion is mediated by PolIII, initiating at nicks catalyzed by gpII of M13 (31). Replication does not require the polymerase activity of PolI (see e.g. (32)) but does require the 5′→3′ exonuclease activity (33,34). This differs in important ways from the theta-replication characteristic of most plasmids.

Here, we present genetic and biochemical characterization of the biological and biochemical properties of two very similar MTases, which interact in unexpected ways with plasmid replication modes.

## MATERIALS AND METHODS

### Bacterial strains, plasmids and reagents

Bacterial strains (all *E. coli*) are listed in Supplementary Table S1, plasmids and phages in Supplementary Table S2 and oligonucleotides in Supplementary Table S3. Cultures were grown in Luria-Bertani (LB) broth or agar on appropriate antibiotics. All RE, DNA MTases, DNA substrates and markers, protein markers, and the PURExpress *in vitro* transcription-translation system were from New England Biolabs (NEB), MA. Q5 ‘Hot Start’ DNA polymerase (M0543, NEB) was used for PCR amplification of genes for cloning. Preparations of plasmid or M13RFI employed Monarch miniprep kits (T1010, NEB). Virion (circular single stranded) DNA from M13 variants was prepared as described in Messing (35). Oligonucleotides were synthesized by IDT, IA. Anti-His-tagged mouse monoclonal antibody (cat. no. 70796) was obtained from EMD-Millipore, USA; Anti-PolI rabbit polyclonal antibody was a gift of Dr. Andy Gardner (NEB); Protein G magnetic beads (S1430) were from NEB. Detection reagents (7003) and secondary HRP derivatives (7074 and 7076) for Western blots were obtained from Cell Signaling Technology, MA.

#### Construction of an M13-sensitive MTase-deficient strain

To study the effect of single stranded MTases M.BceJIII and M.EcoGIX on M13 physiology we designed an unmethylated M13 phage host strain. An F’ factor carrying a Cm^r^ marker gene was conjugated from the donor ER2524 into a recipient, ER2796. The transconjugant was designated ER3661 (Supplementary Table S1).

The phenotype of ER3661 was validated for RM and drug markers: F’, Dam^−^, Dcm^−^, HsdM^−^, EcoK^−^, Mrr^−^, McrA^−^; Kan^R^, Tet^R^, Str^R^, Cm^R^. It was tested for the absence of genomic methylation by digestion with MboI and DpnI restriction endonucleases and for the F' by the ability to propagate M13mp19 phage (36) by plaque formation in soft agar. Inclusion of Xgal and IPTG in the plates enabled clear visualization of the blue plaques, using complementation between the LacZ*alpha* fragment encoded on the phage and the complementing LacZ omega fragment encoded by the F' *lacZΔ*M15.

To use this strain, we designed M13wPB and M13bPB phages to identify m6A modifications in single-stranded virion DNA using the SMRT sequencing approach. This was done by cloning a PacBio blunt end 45 bp phosphorylated adaptor into the SmaI site of M13mp19 (Supplementary Table S3). With the adaptor in one orientation, the phage would package one strand, in the other orientation the complementary strand would be packaged. The adaptor sequence did not inactivate the *lacZalpha* ORF in M13bPB phage; the 183aa alpha-peptide gave blue plaques, similar to the 168aa alpha-peptide of M13mp19. The PacBio adaptor in the other orientation apparently inactivated the LacZalpha-peptide, since no blue plaques were obtained.

### Cloning and expression of M.BceJIII and M.EcoGIX

As described in the introduction, these genes for plasmid-borne MTases were found to be silent in the original hosts (11,14), so plasmid expression constructs were used for the present analysis. All primary cloning and subsequent screening were carried out in ER2683 (Supplementary Table S1). This strain lacks all restriction systems of K-12 and carries an F' overexpressing *lacI*, allowing IPTG regulation of the *lac* promoter (37). The endogenous Dam and Dcm methyltransferases are expressed normally by this strain.

#### Constructs in ColE1 replicons

pAF1 (M.BceJIII), pAF3 (M.EcoGIX) and pEYY63 (pAF3 with inverted origin): To study the functional properties of these unusual MTases, both genes were cloned into pRRS (pUC19 with a variant multiple cloning site). For pAF1, the low-expression *tet* promoter (*tetp*) of pACYC184 replaced the strong *lac* promoter (*lacp*) by design of the PCR oligonucleotide. These plasmids have ColE1 replication origins, *bla* (ampicillin resistance) as a selectable marker and expression under the control of constitutive promoters. The ORFs were PCR amplified from DNA of the native organism with appropriate forward (P1 or P6) and reverse primers (P2 or P7) (Supplementary Table S3) and ligated into the PstI-BamHI sites of pRRS, resulting in pAF1 and pAF3. Additionally, pEYY63 was made: this derivative of pAF3 has a replication origin inverted with respect to the beta-lactamase (*bla*) gene. The origin region (amplified with primers P14 and P15) was ligated to the plasmid backbone (amplified with primers P16 and P17) using a modified Gibson method (38); NEBuilder^®^ HiFi DNA assembly NEB E5520.

#### T7-driven and constitutive expression constructs with a p15A replicon

pAF9 (M.BceJIII) and pAF10 (M.EcoGIX): The inducible T7 promoter expression vector pSAPv6 (a p15A replicon; (39)) was used to add 6xHis-tags to these proteins. PCR with P3, P2 added a 6xHis tag to the M.BceJIII ORF; PCR with P8, P9 added a 6xHis tag and a cleavage site for the protease Factor Xa to M.EcoGIX. These amplicons were digested and ligated to vector NdeI-BamHI sites, creating pAF9 and pAF10.

pAF5 (M.EcoGIX WT) and pAF6 (M.EcoGIX mut): The pSAPv6 derivatives were used as substrates to transfer the tagged genes to an environment enabling low but constitutive expression, using *tetp*. Q5 Hi Fidelity DNA polymerase amplification employed primers P8 and P9 for MTase gene amplification. The p15A plasmid replicon of pACYC184 with *tetp* was amplified with primers P13 and P12. These two amplicons were assembled with the modified Gibson method (38); NEBuilder® HiFi DNA assembly NEB E5520.

#### pSC101 replicon constitutive expression constructs in two orientations

A pSC101 replicon with *tetp*-driven M.EcoGIX expression cassette was constructed by sub-cloning a HindIII-XmnI fragment carrying M.EcoGIX and the Cm^r^ gene from pAF5 (M.EcoGIX) into the HindIII site of pSC101-derived vector pSYX20 (40). This resulted in two plasmids with opposite orientations relative to the replication origin: pAF7 and pAF8.

#### Plasmids with catalytically-inactive MTases

The catalytic DPPY motifs of both M.BceJIII and M.EcoGIX (Supplementary Figure S1) were converted into catalysis-defective mutant variants by introduction of an APPA substitution mutation using reverse PCR. Amplicons were obtained using plasmid templates carrying the wild type MTase allele. Primers P4 and P5 (M.BceJIII) and P10 and P11 (M.EcoGIX) introduced the mutations. The wild type plasmid templates in the amplification reaction were destroyed by DpnI digestion of the Dam-modified (G(m6A)TC) modified backbone to enrich for mutant variants.

Plasmids from candidate colonies were purified using Monarch miniprep kits (NEB, MA) and analyzed by sequencing and restriction digestion. Correct plasmids were identified, and retransformed into strain ER2796, which is deficient in all resident MTases as well as restriction systems (41), selecting Amp^r^ or Cm^r^. Plasmid preparations from this background allowed us to use the RSII Pacific Biosciences sequencing instrument for modification detection and motif deduction.

#### Mobilization plasmids with inverted oriT

Two pESBL variants with 120 bp [INV (*oriT*)] and ∼3.8 kb region [INV *(oriT-nikAB)*] were constructed by Gibson assembly. Three fragments were amplified from pESBL using appropriate primers: p23–p24 for the left fragment, p25–p26 for *oriT* inversion and p27–p28 for the right fragment. The fragments were assembled according to the Gibson protocol along with the pDM4 vector (XbaI/XhoI digested) (42), resulting in pEYY56 allelic exchange vector carrying [INV (*oriT*)]. The second plasmid with [INV *(oriT-nikAB)*] was constructed in two steps. First, two DNA fragments were amplified from pESBL with P23–P29 and P30–P31 primers and assembled into pDM4 (XbaI/XhoI) digested vector resulting in pEYY41 ΔoriT-nikAB allelic exchange plasmids (42). Then the backbone of the plasmid was amplified with P32–P33 primers and *oriT-nikAB* inversion fragment with P34-P35 primers. Two fragments where assembled resulting in the pEYY57 allelic exchange vector with the [INV *(oriT-nikAB)*] mobilization origin. Gene replacements in pESBL were carried out by conventional double crossovers with sucrose sensitivity as the counter-selection (42,43). These mutants were used to carry out transfer efficiency experiments essentially as in (43). Recipient cells were either wild type (MKW278, Cm^R^) or expressing the EcoGIII RM system (cloned in pBAD18Kn, pDM142).

#### Fusion of M.BceJIII and M.EcoGIX to DNA Polymerase I

MTase-PolI fusion constructs (M.BceJIII: pAF13 and M.EcoGIX: pAF14) were created by a three-fragment assembly strategy using NEB Builder HiFi Assembly Master Mix (E2621, NEB, MA) The M.BceJIII and M.EcoGIX genes were amplified (without stop codon) from pSAPv6 derivatives (above) using P18 and P22 or P18 and P21 primers respectively. The *polA* gene encoding DNA Polymerase I (PolI) was amplified from ER2683 genomic DNA using P19 and P20 primers. Three-fragment assembly reactions comprised amplified MTase and *polA* genes and the vector pSAPv6 digested with NdeI-BamHI. The resulting plasmids carry MTase-PolI translational fusions with a linker of 28 amino-acids (DASKDHILQFVIPNRGVTKQLASMTKP). These fusion genes are under the control of a T7 promoter and bear N-terminal 6xHis tags for Ni column protein purification and Western blot detection. The accuracy of the fusion junction was verified by sequencing.

### MTase expression for *in vitro* characterization

His-tagged wild type MTases M.BceJIII (pAF9) and M.EcoGIX (pAF10 and a mutant variant (pAF11) were expressed in T7 Express (ER3081) with IPTG induction. 50 ml LB cultures were grown at 37°C for 4 h to OD_600_ 0.8 and induced with 40 μM IPTG for 4 h at 37°C. 1 ml of crude cell extracts were produced by sonication 6 times for 15″ at 4°C in sonication buffer (20 mM Tris–HCl pH7.5, 50 mM NaCl, 0.1 mM EDTA and 1 mM DTT).

#### PolI loss on purification is accompanied by MTase activity loss

6xHis::M.BceJIII and DNA polymerase I polypeptides were detected by western blotting in active but not in the inactive fraction with monoclonal anti 6xHis-antibody and polyclonal anti-DNA Polymerase I antibody. Detection of MTase was on single-stranded M13mp18 DNA (NEB N4040) with [H^3^]SAM and DNA polymerase activity on sperm whale DNA in the presence of [H^3^]TTP without enzyme, with active enzyme, and with inactive column fractions.

#### Expression and purification for interaction analysis

The His-tagged wild type MTases M.BceJIII and M.EcoGIX and the M.EcoGIX mutant variant were expressed in ER3081 induced with 40 μM IPTG. Protein-protein interactions between MTase and DNA Polymerase I complexes were identified using immunoprecipitation (IP) assays of 6xHis epitope-tagged MTase polypeptides with anti-His-tagged monoclonal antibodies followed by western blot visualization with anti-DNA Polymerase I polyclonal rabbit antibodies (1:5000) or vice versa with IP against DNA Polymerase I with anti-DNA Polymerase I polyclonal rabbit antibodies followed by western blot visualization with anti-His-tagged monoclonal mouse antibodies (1:1000). The IP complexes were collected on protein G magnetic beads (NEB, MA) followed by 3× washing and detection with the HRP western blot detection system (Cell Signaling Technology, MA).

#### Fusion polypeptide purification

Both PolI fusion constructs (pAF13 (M.BceJIII) and pAF14 (M.EcoGIX)) were transformed into T7 Express and induced with 40 μM IPTG in LB overnight at 16°C. Three liters of these induced cultures expressing fusion proteins were carried through purification as described below.

### Immunoprecipitation and western blots

The MTase polypeptides were precipitated with anti-6xHis mouse antibodies bound to Protein G magnetic beads (NEB, MA), washed 3 times in sonication buffer, eluted with 50 μl SDS loading buffer and separated on 10–20% SDS PAGE. Similarly, DNA Polymerase I was precipitated with anti-PolI rabbit antibodies bound to Protein G magnetic beads, followed by similar washing, elution and SDS-PAGE separation. The Western blots were developed with either anti-DNA Polymerase I rAb or anti-6xHis-tag mAb according to a standard Western blot detection protocol (CST, MA).

### Protein purification and enzymatic assays

His-tagged MTase polypeptides of wild type M.BceJIII and M.EcoGIX and the M.EcoGIX mutant variant (Supplementary Figure S2) and their *in vivo* complexes (see Results) were purified by affinity chromatography on a Ni-HiTrap column (17524801) followed by ion-exchange chromatography on HiTrap Heparin HP (17040701) and HiTrap QHP (17115401) columns from GE Healthcare, USA using the AKTA 9000 protein purification system. The resulting proteins were resolved on 10–20% SDS-PAGE and visualized either by Coomassie dye stain or by western blot assays with anti-His-tagged monoclonal antibodies.

The PolI fusion proteins, 6xHis::M.BceJIII::PolI and 6xHis::Xa::M.EcoGIX::PolI were carried through three steps of purification: 5 ml HiTrapNi with step elution by 250 mM imidazole; 5 ml HiTrapHepHP, and 5 ml HiTrapQHP ion exchange column chromatography, both with NaCl gradient elution (50–800 mM) in 20 mM Tris–HCl pH7.4, 1 mM DTT and 0.1 mM EDTA buffer. About 1 mg of each the purified fusion proteins was concentrated to 1 mg/ml in storage buffer A (10 mM Tris-HCl pH7.4, 1 mM DTT, 0.1 mM EDTA, 50 mM KCl, 50% glycerol). These fractions were analyzed for DNA MTase activity on single-stranded M13mp18 DNA (NEB N4040) in the presence of [H^3^]SAM and for PolI activity on sonicated sperm whale DNA in the presence of [H^3^]TTP. The purity of the final proteins was analyzed on 10–20% SDS PAGE using western blots against anti-DNA Polymerase I rabbit antibodies and anti-6xHis mouse antibodies for fusion protein detection.

Qualitative assessment of MTase activity employed a methylase protection assay on plasmid DNAs. Substrate DNAs were methylated by the enzymes with cold S-adenosylmethionine (SAM) (80 μM), then challenged by the RE HincII (GTYR**A**C). Methylation of the highlighted adenine residue (on either strand) will block cleavage.

Quantitative tests of MTase activity employed radioactive [H^3^]SAM (0.66 μM) (Perkin Elmer, MA), MTase and DNA, followed by separation of labeled DNA from unincorporated [H^3^]SAM on Monarch mini-prep columns (T1034, NEB). [H^3^]-Methyl groups incorporated into the substrate DNA were detected by scintillation counting (Tri-Carb 2900 Analyzer (PerkinElmer)).

In addition, [H^3^]-methyl labeled DNA fragments were visualized by fluorography. Fragments were separated on appropriate agarose or 20% PAGE gels in TBE buffer, transferred to Hybond^TM^-N^+^ (Amersham, NJ) positively charged membranes, sprayed with EN^3^HANCER (Perkin Elmer) and visualized with ECL Hyperfilm™ (Amersham, NJ). DNA markers for fluorography were made by modification of NEB N3012 (Lambda HindIII digest) or NEB N3233 (Low Molecular Weight DNA Ladder) with the nonspecific A-DNA MTase, M.EcoGII (NEB M0603). Note that M.EcoGII is active on both single- and double-stranded DNA.

#### MTase activity on single- or double-stranded substrates

The MTase activity from PURExpress and purified protein fractions from *E. coli* 3081-expressed extracts were tested on single-stranded (M13mp18 virion, NEB N4040) and double stranded DNA (M13mp18 RFI, NEB N4018) substrates. For oligonucleotide assays, two differentially labeled (5′TAM and 5′FAM) oligonucleotides were used as single-stranded or duplex substrates in a H^3^-SAM MTase reaction.

### LC–MS analysis

#### M13 virion DNA

10 ml LB cultures were inoculated with a single M13bPB phage and incubated overnight at 37°C. Single-stranded DNA was purified (35) and digested to nucleosides with a nucleoside digestion mixture (NEB M0649) and analyzed on LC–MS. The level of m6A modified nucleosides (as a % of total A) was reported.

#### 6xHis::M.BceJIII interacting proteins

Protein digestion: Protein samples from HiTrapNi and HiTrapQHP (fractions containing active 6xHis::M.BceJIII) and HiTrapHeparinHP (fractions containing inactive 6xHis::M.BceJIII) were diluted to 1 μg/μl in 10 mM Tris–HCl, 1 mM DDT, 0.1 mM EDTA 50 mM KCl pH7.4 50% glycerol. Protein samples were reduced with 5 mM DTT (Sigma) at 60°C for 20 min. After allowing samples to cool, they were alkylated with 10 mM iodoacetamide (Sigma). Solid urea was added to 4M final concentration (44). Protein samples were digested with trypsin (NEB) at 37°C, 1:50 E:S ratio overnight. The digestion reaction was quenched with TFA at 0.5%.

MudPIT: Digested proteins were directly loaded on a triphasic capillary column as described (45). LC buffers were: buffer A (5% ACN/0.1% formic acid), buffer B (90% ACN/0.1% formic acid) and salt buffer solution C (500 mM ammonium acetate/5% ACN/0.1% formic acid). The triphasic column was then connected to an analytical column of a 100-μm i.d. capillary prepacked with 25 cm of 3-μm material Reprosil-Pur C18 AQ (New Objective). A Proxeon II LC nanoflow pump was used to fractionate peptides with a reversed phase gradient followed by 7-salt steps applied using the autosampler. The following sequence of salt steps was used for peptide analysis: load (buffer A), 20% C, 40% C, 100% C, 95% salt–5% B, 90% salt–10% B, 85% salt–15% B, followed by a last step of 80% salt/20% B. For each salt step, eluted peptides from the microcapillary fritless column were directly electrosprayed into a linear ion trap-orbitrap mass spectrometer (LTQ Orbitrap XL) and MS/MS were acquired during separation (46).

Database searches: Acquired MS/MS spectra were searched against an *E. coli* protein sequence database supplemented with target protein sequences using PEAKS 8.5 (Bioinformatics Solutions Inc). Sequences of trypsin, keratin and contaminants were added to the database. The protein FDR was set at 1%.

### DNA sequencing and bioinformatics

PCR fragments and plasmid DNA constructs were sequenced on the ABI DNA sequencer with a set of appropriate sequencing primers. The *in silico* plasmid designs were assembled using Laser Gene).

The SMRT next generation sequencing technology of Pacific Biosciences Inc. allowed us not only to verify the accuracy of our constructs, but also to derive the modification status of the DNA at the same time using the kinetic signature of DNA polymerase on the template during the reaction.

SMRTbell template libraries were prepared using the PacBio protocol, adapted for NEB library construction components. In brief, plasmid and/or genomic DNA samples were sheared to an average size of ∼2 kb using the Clear miniTubes protocol (Covaris; Woburn, MA, USA), repaired by PreCR treatment, end repaired and ligated to hairpin adapters. Incompletely formed SMRTbell templates were digested with a combination of Exonuclease III and Exonuclease VII (New England Biolabs; Ipswich, MA, USA). Genomic/plasmid DNA fragments and SMRTbell library qualification and quantification were performed on the Qubit fluorimeter (Invitrogen, Eugene, OR) and 2100 Bioanalyzer (Agilent Technologies, Santa Clara, CA, USA). SMRT sequencing was carried out on the PacBioRSII (Pacific Biosciences; Menlo Park, CA, USA) using standard protocols for small insert SMRTbell libraries. Sequencing reads were processed, mapped and assembled with the Pacific Biosciences' SMRT Analysis pipeline (http://www.pacbiodevnet.com/SMRT-Analysis/Software/SMRT-Pipe) using the re-sequencing protocol (47).

Rough estimates of replicon copies per host chromosome were obtained by sequencing preparations of plasmids or M13 RFI. In these preparations, generally residual chromosomal DNA is still present; the fraction of total DNA derived from the chromosomal contamination is generally higher for low-copy than for high-copy plasmids. Accordingly, the read number matching the plasmid sequence was divided by the read number matching the chromosomal sequence. This ratio probably overestimates the actual copy number, since plasmid preparations selectively recover small covalently closed circular DNA using rapid alkaline denaturation and renaturation. The method shears chromosomal DNA.

Modification at each nucleotide position was measured as kinetic variations (KVs) in the nucleotide incorporation rates; methylated motifs were deduced from the KV data (4,48,49); fraction of particular motifs modified is also reported by the protocol.

## RESULTS

### *In vivo* m6A modification acts only on the leading strand in replication

**Figure 1.**
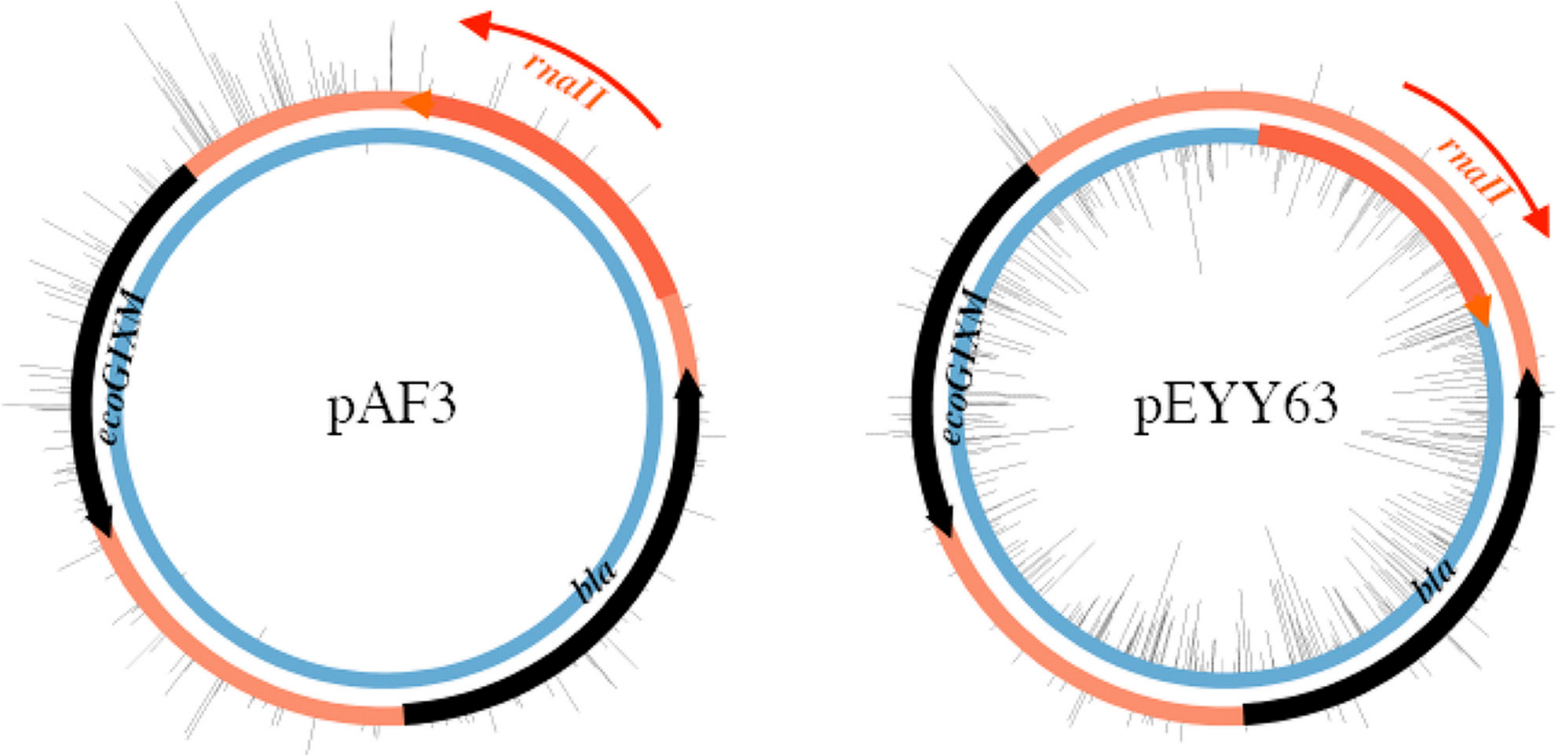
The ColE1 *ori* orientation determines which strand is modified by M.EcoGIX. Black ticks: observed sites of m6A modification. Black arrows: coding sequences for beta-lactamase (*bla*) and the MTase (*ecoGIXM*). Thick orange arrow: the origin of replication (*ori*), determined by the *rnaII* nucleotide sequence. RNAII primes leading strand DNA synthesis in the direction of the thin orange arrow.

The unusual plasmid-borne MTases M.EcoGIX and M.BceJIII were cloned in the constitutive expression vector pRRS under control of either *lacp* or *tetp*, then propagated in the methylation-deficient host, *E. coli* ER2796. SMRT sequencing of these plasmid DNAs revealed degenerate m6A modification at the motif SAY (where S = C or G and Y = C or T), but almost exclusively on one strand (Figure 1) (11,14). Oddly, no m6A modification was detected on genomic DNA carried along in the DNA preparation.

To explore the properties of this unusual strand-specific plasmid methylation, we inverted the ColE1 origin of replication in pAF3, creating pEYY63. Surprisingly, the m6A modification pattern switched strands, suggesting that MTase activity *in vivo* is associated with the plasmid replication process, specifically modifying the leading strand (Figure 1). Again, genomic DNA was free of m6A.

### MTase catalytic mutants and *in vivo* sensitivity to modification-dependent EcoKMrr restriction

#### Structural modeling analysis using the Phyre2 server

Model prediction with Phyre2 (http://www.sbg.bio.ic.ac.uk/∼phyre2/html/page.cgi?id=index) identified M.BceJIII and M.EcoGIX (Supplementary Figure S1 panels A and B) as highly homologous proteins of the beta family of DNA MTases. This assignment was confirmed by amino-acid sequence alignment and identification of the expected conserved DNA MTase motifs (Supplementary Figure S1 panel C) (50,51). Based on the modeling data, the DPPY catalytic motif IV was converted to APPA in both MTases to create catalytically defective variants of both enzymes.

#### *In vivo* sensitivity to modification-dependent restriction

The modification-dependent restriction enzyme (MDRE) EcoKMrr shows context-dependent restriction of m6A- and m4C-modified DNAs (52,53). Wild type (WT) and mutant alleles of M.EcoGIX and M.BceJIII were tested for sensitivity to this restriction *in vivo*: reduction in transformation efficiency by a restricting strain (ER1516 Mrr^+^) relative to an isogenic permissive strain (ER1969, Mrr^−^) (Supplementary Table S4). Three different vector replicon configurations were used: those with a p15A replicon are found at lower copy (∼15/cell) than the ColE1 replicon (without *rop*, encoding a protein regulator; ∼100/cell). The pSC101*ts* replicon, ∼20/cell, is similar to p15A. The promoter used for expression also differed: the low constitutive *tetp* for p15A, or an unrepressed *lacp* for the ColE1 derivatives used here.

All plasmids carrying WT MTase alleles were restricted 10–10 000-fold (EOT column) except pAF7 (to be discussed further below), while none of the plasmids carrying mutated alleles were restricted as judged by the EOT ∼1. Low EOT thus shows that both the presence of m6A modification confers sensitivity to Mrr restriction, and the mutation design had successfully eliminated the sensitivity and presumably modification. Five colonies surviving the strongest restriction (row 1) were sequenced; all exhibit mutations in the plasmid MTase gene, consistent with incompatibility between the MTase and the MDRE EcoKMrr.

### Plasmid replication mode determines methylation activity and affects plasmid copy number

To study the effect of different replicons on MTase activity *in vivo*, wild type and mutant alleles of M.BceJIII and M.EcoGIX were cloned into ColE1, p15A and pSC101 replicons. The first two share a replication initiation program that depends on host RNAP transcription and DNA Polymerase I (20). They differ in copy number and are compatible. The third, pSC101, depends on a plasmid-specified initiator protein and host DNA polymerase III. Thus, we can separate copy-number effects from effects of replication mode on modification. Both approximate relative copy number and modification state can be determined from the same data on the PacBio RSII.

#### MTase effects on copy number

Plasmid copy number was estimated as plasmid read coverage divided by chromosome coverage. Each plasmid construct was transformed into the methylation-deficient strain *E. coli* ER2796, purified and analyzed by gel electrophoresis of restriction digests, and by sequencing on the PacBio RSII instrument. Plasmid copy number was estimated by quantitating reads from plasmid 2kb SMRT sequencing libraries that mapped to the plasmid reference sequence and then normalizing by the number of reads that mapped to the chromosome.

These MTases impose costs on the susceptible replicons, whether the genes for them are present on the same molecule (*in cis*) or a different one (*in trans*). Carriage of WT M.EcoGIX or M.BceJIII by ColE1 yields a lower copy number for WT than for corresponding mutant alleles (Table 1A, compare columns 1 and 3; 2 and 4). M.EcoGIX affects p15A similarly (Table 1A, compare columns 5 and 6). Curiously, this effect acts *in cis* to penalize the susceptible replicon: in doubly-transformed cells the ColE1 plasmid was present at much lower copy number than the pSC101 plasmid when both carried WT MTase alleles (Table 1B, column 8), but rose to the usual higher copy when it bore a mutated allele (Table 1B, column 9, even in the presence of pSC101-borne WT still capable of modifying ColE1 (Table 1B, column 8) but not itself Table 1B, column 9) (see further below).

**Table 1.** Copy number and modification levels.

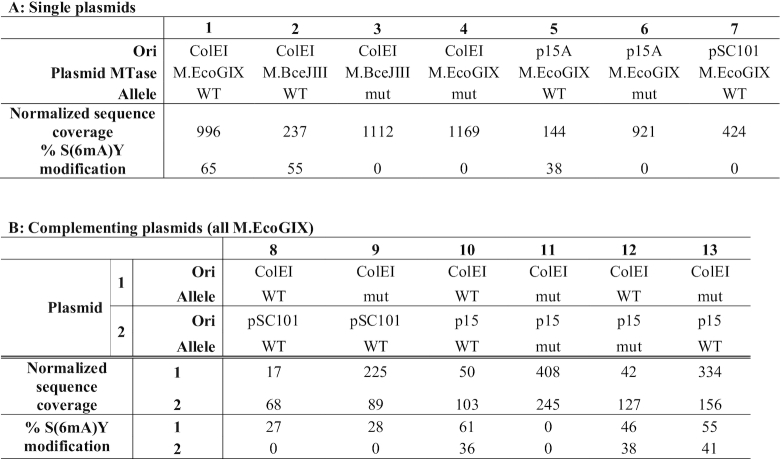

MTase: native MTase sequence cloned into the vector. Allele: WT, native wild type; mut: quadruple substitution APPA in the active site. Normalized Sequence Coverage: reads mapping to the plasmid, relative to chromosomal reads present; % of SAY sites modified. 100% of modified bases detected were in SAY sites. Panel A, singly transformed cells with lane 1: pAF3; lane 2: pAF1; lane 3: pAF2; lane 4: pAF4; lane 5: pAF5; lane 6: pAF6; lane 7: pAF7. Panel B, doubly-transformed cells with lane 8: pAF3 + pAF7; lane 9: pAF4+pAF7; lane 10: pAF3+pAF5; lane 11: pAF4+pAF6; lane 12: pAF3+pAF6; lane 13: pAF4+pAF5.

#### ori-dependence of modification state

The same reads were used for SMRT motif and modification analysis. This enabled quantification of modification level using the kinetic signature of m6A on the modified plasmid. Both ColE1 (Table 1A, columns 1, 2, 3, 4) and p15A (Table 1A, columns 5, 6) replicons support m6A modification at SAY (Table 1A, columns 1, 2 and column 5). In contrast, the pSC101 replicon did not support SAY modification at all (Table 1A, column 7).

Strikingly, this dependence on replication mode is dominant. In doubly transformed cells carrying WT MTase alleles on both a ColE1 and a pSC101 plasmid, only the ColE1 plasmid is modified (Table 1B, column 8). The protein can act *in trans*: in doubly transformed cells with a mutated MTase allele *in cis* on a ColE1 plasmid and a WT MTase allele *in trans* on a pSC101 plasmid, the ColE1 plasmid was modified at the SAY motif (Table 1B, column 9) even though the pSC101 plasmid itself was not modified.

We deduce that the WT MTase can act *in trans*, but action is dependent on a feature common to ColE1 and p15A and distinct from pSC101 and the chromosome. In view of the orientation-dependence of modification pattern, the role of DNA Polymerase I is of interest.

#### Genetic confirmation of polA-dependence of the copy number effect of M.EcoGIX

Both ColE1 and p15A replicons depend on PolI during replication, while pSC101 does not. Accordingly, a *polAts* allele was used to explore whether M.EcoGIX MTase toxicity depended genetically on the state of PolI. PR602 (an RR1 derivative carrying the *ts* allele of *polA* from PR597 (20)) was used for this purpose. The p15A replicon was maintained in the *polAts* mutant at permissive temperature, although the ColE1 derivative could not be established. For the p15A derivative, the plasmid copy number dropped dramatically when shifted to the nonpermissive temperature (Supplementary Figure S3). The drop was more acute with the native M.EcoGIX allele than with the APPA catalytic mutant. The pSC101 replicon was unaffected, consistent with reports (54) (data not shown).

The level of SAY plasmid modification was not estimated. GATC sites are a subset of SAY sites and were likely fully modified by the wild type Dam methylation present in the host PR602. However, no sequences other than SAY motifs were detected by SMRT sequencing.

### M.BceJIII and M.EcoGIX MTases suppress M13 phage replication *in vivo*

The M13 filamentous phage has a different replication program than any of the plasmids above. Initiation requires the chromosomal primase, DnaG, followed by a PolIII-dependent two-phase mechanism to generate circular RFI. This phase requires the 5→3 exonuclease activity of PolI (33), but not the polymerization activity (see, e.g. (32)). The third, rolling circle phase produces single stranded DNA packaged into virions (infectious viral particles) that are then secreted through the cell wall.

An M13-sensitive, methylation-defective host, ER3661, was used to propagate M13 in the presence of plasmid-borne MTase alleles. We find a dramatic effect of both WT MTases on RFI copy number, which is abolished for the catalytic mutants (Table 2A). Other replicons in the same cell respond as expected (MTase expression plasmids and resident F factor). As observed above, WT MTase reduces the copy number of its own plasmid, but the effect on M13RFI is much larger. The modification of M13 RFI and the plasmids confirm the activity of the resident MTases. As might be expected, the conjugal F factor is not modified. In a separate experiment, virions from the supernatant were examined (Table 2B); these samples recapitulate the RFI result, with poor yields in the presence of the WT MTases. The virion DNA that is produced is heavily methylated, suggesting that the phage produced are not liberated by subpopulations lacking the MTase gene or plasmid.

**Table 2.** M.BceJIII and M.EcoGIX MTases, but not inactive variants suppress M13 replication and virion production *in vivo*.

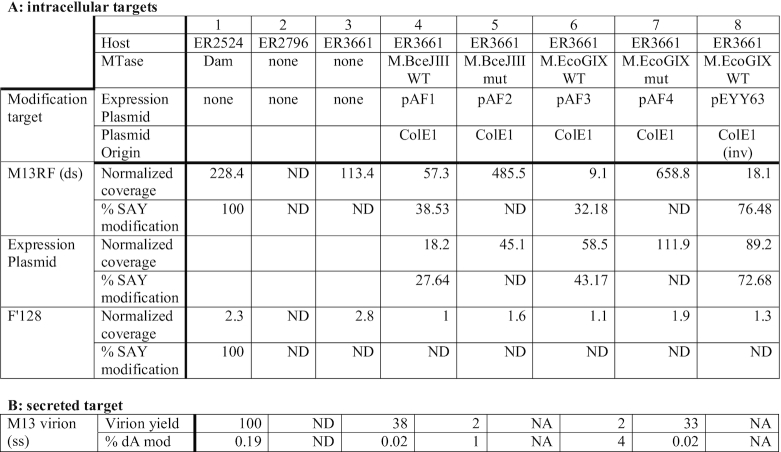

ND: not detected; NA: not applicable. Table 2A: Coverage: Number of PacBio reads mapped to reference sequences; % modification: SAY sites modified/SAY present x 100, except control host ER2524 column 1, Dam sites (GATC) modified/Dam sites present x 100. Expression plasmids were pAF1 (bceJIIIM WT); pAF2 (bceJIIIM mut); pAF3 (ecoGIXM WT); pAF4 (ecoGIXM mut); pEYY63 (ecoGIXM WT Ori inv). Table 2B: virion DNA was prepared by infection of ER2524 (Dam-modified host), ER2796 (non-modifying F- non-host), ER3661 (non-modifying host), and ER3661 transformed with pAF4 (ecoGIXM mut), pAF3 (ecoGIXM WT) or pAF1 (bceJIIIM WT); M13 virion yield was estimated from Qubit reads of preparations and expressed relative to the Dam+ control host ER2594 taken as 100%. Fraction of modified dA residues in the preparations was determined as by LC-MS described in Materials and Methods.

### *In vitro* characterization of His-tagged M.BceJIII and M.EcoGIX

#### Defining the substrate in partially-purified preparations

All attempts to purify native, untagged MTase proteins beyond crude extract failed; activity was found with single-stranded M13 (not shown) but was lost after two columns (DEAE, then Heparin). Accordingly, wild type and mutant variants of M.BceJIII and M.EcoGIX were isolated with 6xHis tags from plasmids listed in Supplementary Table S2, constructed as in Materials and Methods. These tagged variants were expressed either *in vitro* with the PURExpress transcription-translation system (Supplementary Figure S2 panel A) or *in vivo* in NEB T7 Express followed by purification on AKTA 9000 (Supplementary Figure S2 panel B). Again, activity was lost after the third column (Supplementary Figure S2; see Materials and Methods).

#### Single-strand preference with a complex substrate

**Figure 2.**
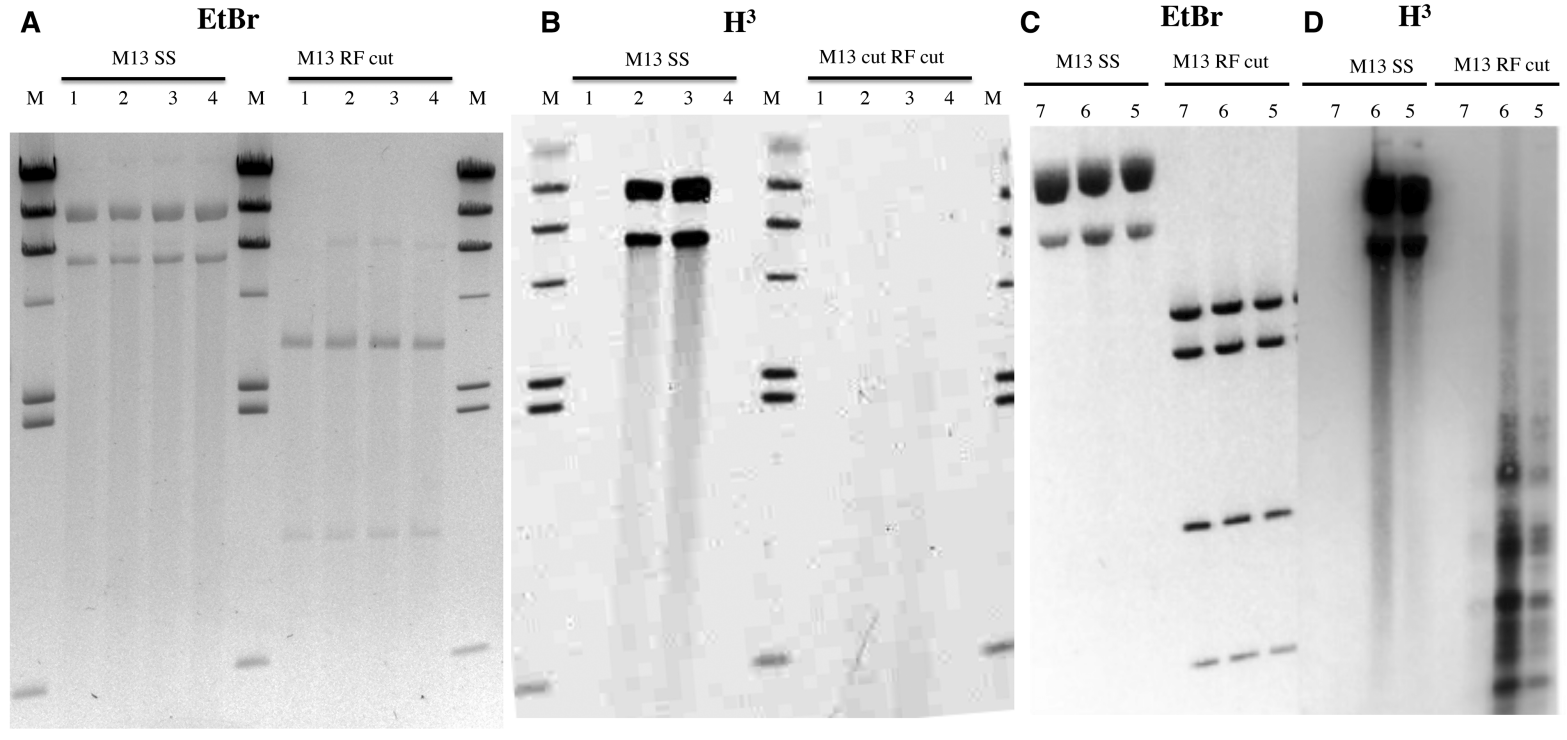
MTase activity requires single strands. Panels (**A**) and (**C**): M13 substrates stained with ethidium bromide. Panels (**B**) and (**D**): fluorograms of modification reactions using [H^3^]SAM. M13 SS: virion DNA substrate. M13 RF cut: DS replication intermediate RFI was digested following the labelling reaction for visual simplification; NdeI (Panels A and B) or NdeI+BamHI (Panels C and D). The substrates were treated with MTase proteins obtained with PURExpress *in vitro* transcription-translation (Panels A and B) or were partially-purified (Ni-NTA purification) proteins synthesized *in vivo* (Panels C and D). Lanes 1) empty pSAPv6 vector, 2) M.BceJIII WT (pAF9), 3) M.EcoGIX WT (pAF10) and 4) M.EcoGIX APPA variant (pAF11). H^3^ radiolabeled markers (M) are HindIII digested lambda DNA modified at A by M.EcoGII.

The tagged WT proteins from *in vitro* expression (‘PURExpress’ MTases) exhibited activity visualized by fluorography (Figure 2) with a single-stranded but not a double-stranded substrate (M13mp18 virion or RFI; Figure 2B). When the same 6xHis-tagged WT M.BceJIII and M.EcoGIX were purified from crude extracts on Ni-NTA beads, single-stranded M13mp18 DNA substrates were modified (Figure 2D). With the Ni-NTA eluted fractions and double-stranded M13mp18, some activity was found in small fragments (Figure 2D). Since other *E. coli* enzymes derived from the crude extract are still present in these fractions, we speculate that the pattern of H^3^-labeled DNA may represent action at single-strand gaps created by extraneous activities. Alternatively, this could represent off-target (‘star’) activity due to high enzyme concentration. No RNA-modifying activity was found with the *in vitro* expressed His-tagged MTases (Supplementary Figure S4); these samples were active on M13 single-stranded DNA (Supplementary Table S5).

As with the native proteins, all attempts to further purify active His-tagged MTase proteins from crude extracts failed (Supplementary Figure S2). Ni-NTA imidazole elution fractions and HiTrap QHP fractions retained activity. Any additional purification steps using a Heparin column led to >90% loss of MTase activity for both M.EcoGIX and M.BceJIII. We did obtain high purity but inactive MTase polypeptides.

### DNA polymerase I (PolI) may be a component of active M.BceJIII

#### LC–MS analysis of active and inactive MTase fractions

The MTase proteins may work as a part of a weakly-associated complex with loss of an essential component abolishing MTase activity *in vitro*. To gain insight into possible factors, we used LC–MS analysis of 6xHis::M.BceJIII active and inactive MTase protein fractions described above to examine the composition of the active MTase complexes. 200 μg of proteins from active and inactive fractions were analyzed by LC–MS. A summary of 6xHis::M.BceJIII interacting polypeptides detected by LC–MS is presented in Supplementary Table S6.

The increase in counts of polypeptides derived from 6xHis::M.BceJIII (51 in the active fraction [column B] to 247 in the inactive fraction [column C]) indicates a 5-fold increase in purity after the heparin column. As a negative control (column A), we included mock-purification fractions (T7 Express carrying empty vector pSAPv6) carried through the Ni-imidazole column. A list of potential interacting polypeptides was defined as those absent in the negative control (column A), present in the active fraction (column B), then lost in the inactive fraction (column C). Thirty-seven interacting polypeptides were detected using these criteria. They fell into two major categories. The 16 blue rows identify parts of the translational machinery. These may indicate that the N-terminally His-tagged polypeptides of M.BceJIII were undergoing active translation, thus retaining ribosomes on Ni columns. The 19 yellow rows identify potential interacting proteins that have not been analyzed yet.

The one red row identifies the presence of DNA Polymerase I. Identification of PolI was illuminating, since such an interaction could shed light on our *in vivo* data. Specifically, *in vivo* we found that these MTases act on those plasmids that share a requirement of PolI for plasmid replication, but do not act on plasmids with a pSC101 replicon, which is PolI-independent.

#### Western blot and reciprocal immunoprecipitation characterization of active complexes

**Figure 3.**
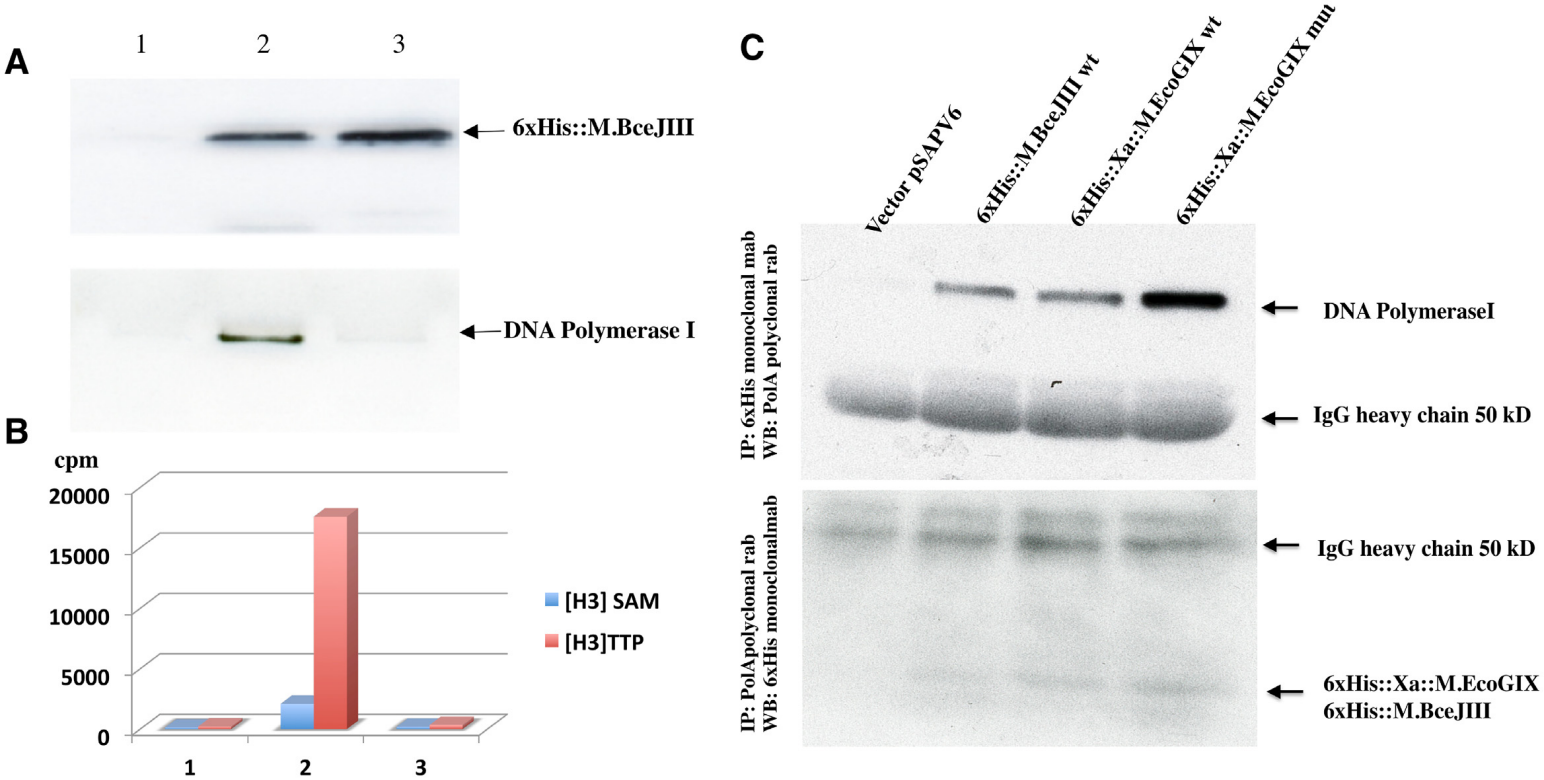
Immunologic and enzymatic detection of PolI in active MTase fractions from *in vivo* expression. Panel (**A**): Anti-His-tag detects 6xHis::M.BceJIII from pAF9 in both active and further-purified inactive fractions (top row), but anti-PolI detects the polymerase only in the MTase active fraction (bottom row). Panel (**B**): MTase action and nucleotide incorporation by these fractions. Blue bars: single-stranded M13mp18 DNA modified with [H^3^]SAM; red bars: sperm whale DNA labeled by [H^3^] dTTP (DNA polymerase activity) measured without enzyme (1) or with active (2) or inactive (3) MTase column fractions. Panel (**C**): Reciprocal immunoprecipitation assays recover PolI and His-tagged MTases together. Top panel: Western detection of PolI by anti-PolI of MTase tagged anti-His IP. Bottom panel: Western detection of His-tagged MTase anti-His of anti-PolI IP. *In vivo* expression employed pAF9 (M.BceJIII), pAF10 (M.EcoGIX WT) and pAF11 (M.EcoGIX mut).

To pursue the role of PolI by an independent method, we tested active and inactive fractions of 6xHis::M.BceJIII with western blots for detection by anti-6xHis-tag mouse antibodies (Figure 3, Panel A, top gel) and anti-DNA Polymerase I rabbit antibodies (Figure 3, Panel A, bottom gel). MTase was detected in both fractions, while PolI was detected only in the active fraction.

The same fractions were tested for modification and polymerase activity. DNA polymerization (dTTP incorporation) was detected only in the fraction with active MTase (Figure 3, Panel B).

To confirm the presence of DNA Polymerase I and MTase in the same complexes we performed reciprocal immunoprecipitation (IP) assays from crude extracts of ER3081 with empty vector or with wild type or catalytically-defective M.EcoGIX and M.BceJIII alleles (Figure 3, Panel C). IP of 6xHis epitope-tagged MTase polypeptides employed anti-His monoclonal antibodies, with Western blot visualization using anti-PolI polyclonal rabbit antibodies, while IP of Pol I employed the anti-PolI rabbit antibodies and western blot visualization with the anti-His monoclonal.

Precipitation of His-tagged MTase proteins recovers PolI (Figure 3, Panel C, top), while precipitation of PolI recovers the His-tagged MTase proteins (Figure 3, Panel C, bottom). The mutant variant of M.EcoGIX may have a stronger interaction with PolI or form a more stable complex than wild type MTases, as judged by the more-intense band of PolI recovered in that lane.

### Fusion of M.EcoGIX and M.BceJIII MTase with PolI enables purification of MTase activity

**Figure 4.**
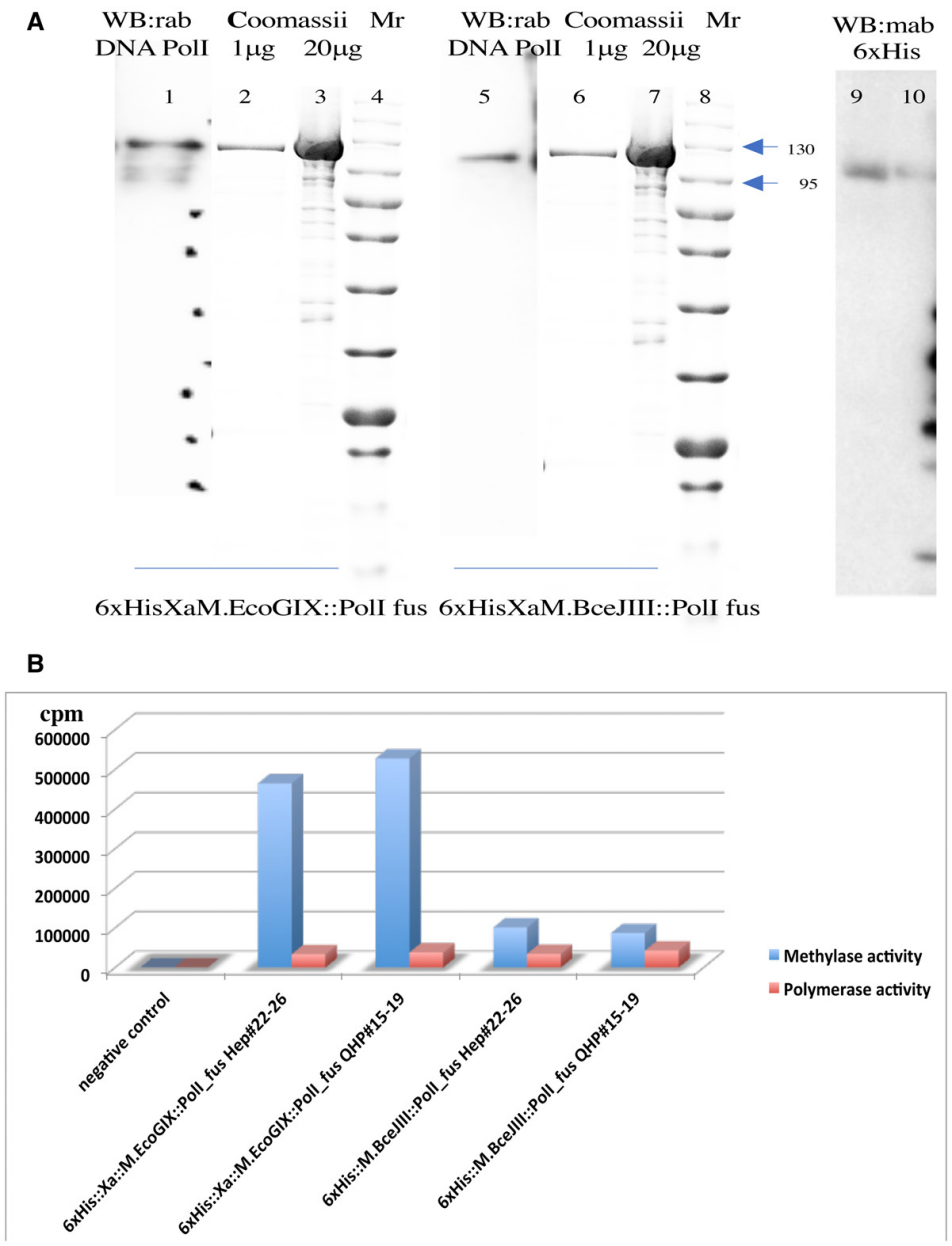
Polymerase and MTase activities copurify when domains are fused. Panel (**A**): Size and purity of fusion proteins. For each MTase, both of the immunoreactive components of the MTase-PolI fusion proteins run at the same position, and comigrate with the Coomassie-stained purified proteins. Western blot (lanes 1, 5, 9 and 10) detected 1 μg of MTase-PolI fusion proteins; Coomassie (lanes 2, 3, 6, 7) visualized 1 μg or 20 μg of the same fractions. Western blots were probed separately with anti-Pol1 rabbit polyclonal or anti-6xHis (detecting the MTase) monoclonal antibodies and developed with horseradish peroxidase-labeled antirabbit or antimouse following kit instructions as detailed in Material and Methods. Dots on lane 1 correspond to the position of protein markers after Western blotting. The bands at the side of lane 10 are spillover from the adjacent lane, which were control 6xHis tagged proteins from a PurExpress extract. Panel (**B**): Activity copurification through two columns. Pooled HiTrapHepHP (#22–26) and HiTrapQHP (#15–19) protein fractions were tested for MTase activity on single-stranded M13mp18 DNA in the presence of [H^3^]SAM and for DNA-polymerase activity on sonicated sperm-whale DNA in the presence of [H^3^]TTP.

We could not restore MTase activity to heparin-purified MTases by adding back purified PolI or its Klenow fragment. To explore possible coordinated action between the two components, we created translational fusions between the MTase and PolI genes (see Materials and Methods). Both constructs (pAF13 (M.BceJIII::PolI) and pAF14 (M.EcoGIX::PolI)) were transformed into T7 Express and induced with 40 μM IPTG in LB overnight at 16°C. Three liters of these induced cultures expressing fusion proteins 6xHis::M.BceJIII::PolI and 6xHis::Xa::M.EcoGIX::PolI were carried through three steps of purification: 5 ml HiTrapNi with step elution by 250 mM imidazole; 5 ml HiTrapHepHP, and 5 ml HiTrapQHP ion exchange column chromatography, both with NaCl gradient elution (50–800 mM) in 20 mM Tris–HCl pH7.4, 1 mM DTT and 0.1 mM EDTA buffer. About 1 mg of each the purified fusion proteins was concentrated to 1 mg/ml in storage buffer A (10 mM Tris–HCl pH7.4, 1 mM DTT, 0.1 mM EDTA, 50 mM KCl, 50% Glycerol). The purity of the resulting fusion proteins (130 kD for 6xHis::M.BceJIII::PolI and 132 kD for 6xHis::Xa::M.EcoGIX::PolI) was analyzed on 10–20% SDS PAGE using Western blots against anti-DNA Polymerase I rabbit antibodies and anti-6xHis mouse antibodies for fusion protein detection (Figure 4, Panel A).

These fusions did indeed exhibit both MTase and DNA polymerization activities co-purifying over three columns. The final fractions were analyzed for DNA MTase activity on single-stranded M13mp18 DNA in the presence of [H^3^]SAM and for PolI activity on sonicated sperm-whale DNA in the presence of [H^3^]TTP (Figure 4, Panel B). The DNA MTase activity of the 6xHis::Xa::M.EcoGIX::PolI fusion was about 5 times higher than that of the 6xHis::M.BceJIII::PolI fusion, while the DNA Polymerase I activity of the two fusion proteins was similar.

#### MTase-Pol fusions modify oligonucleotide single-strand substrates

Purified MTase-PolI fusions were also tested on single- and double-stranded oligonucleotides (Supplementary Figure S5). Complementary 5′-FAM and 5′-TAM-labeled oligonucleotides and their annealed duplexes were incubated in the presence of H^3^SAM and active MTase. Both single-stranded substrates were labeled, but the double-stranded oligonucleotide created by annealing the two was not labeled under the same conditions. H^3^ incorporation into single strands correlated with the number of SAY motifs in substrate DNA. Therefore, single stranded DNA substrates are preferred for the MTase activity of the fusions. This did not require additional nucleotides.

#### Coordinated action of two activities on primed substrates

**Figure 5.**
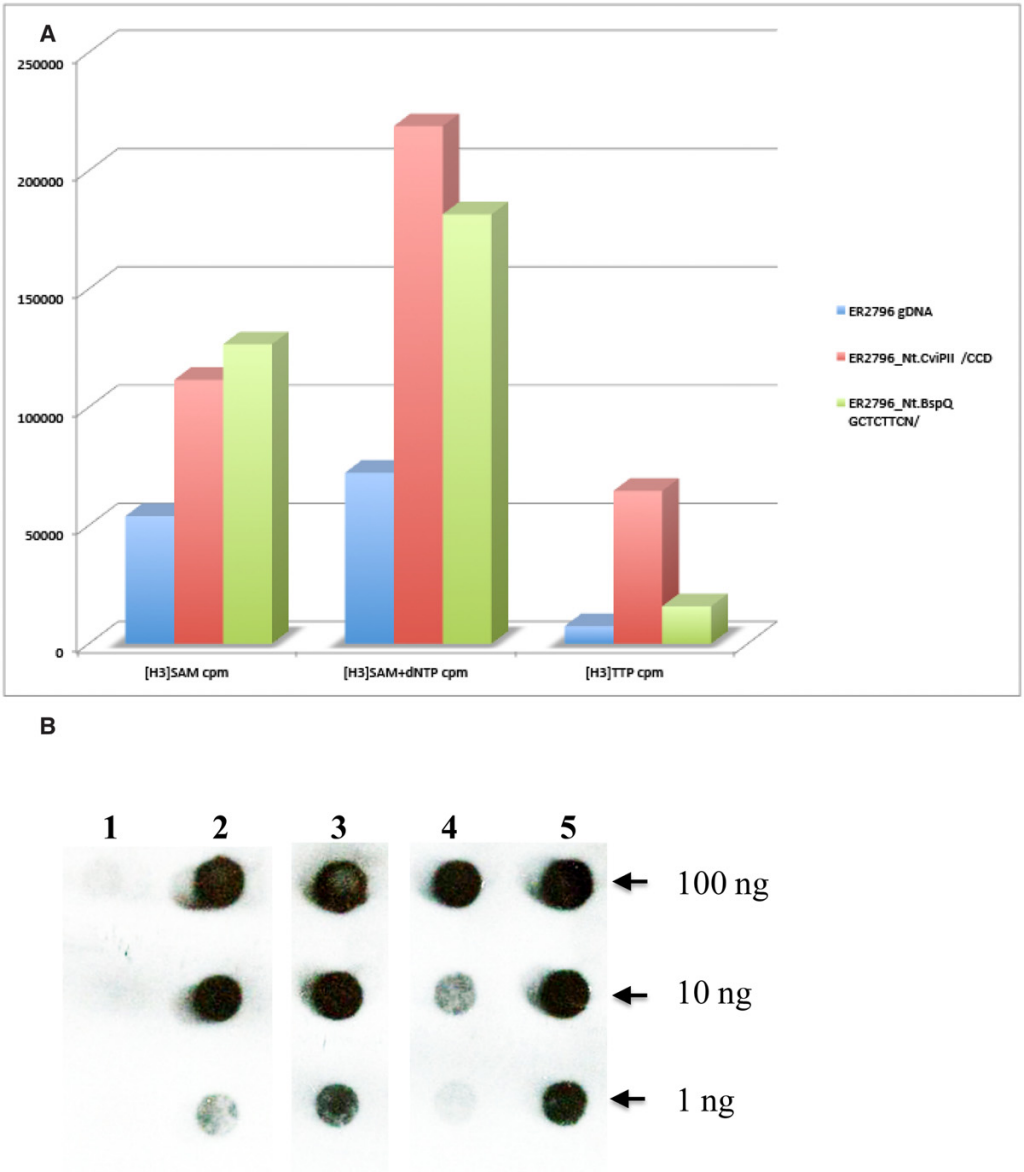
Nicks stimulate both MTase and polymerase activities of the M.EcoGIX::PolI fusion. Panel (**A**): MTase activity was measured with [H^3^]SAM alone (left set of bars) or with cold dNTP to enable nick translation (middle set). DNA polymerase activity was measured with [H^3^]TTP (right set). The gDNA substrate (unmethylated ER2796 as isolated) has preexisting nicks that provide priming sites for polymerization (blue bars). Additional priming sites were added using nicking enzymes, either with a frequent site (Nt.CviPII |CCD, red bars) or with a rarer site (Nt.BspQI, GCTCTTCN|, green bars). Panel (**B**): Immunologic detection of m6A modification using anti-m6A monoclonal rabbit antibodies. A M.EcoGIX::PolI nick-translation reaction with gDNA as in Panel A was spotted and developed with antibody. (1) ER2796 gDNA, no enzyme control; (2) ER2796 gDNA nicked with Nt.BspQI (671 sites per genome); (3) nicked with Nt.CviPII (438,784 sites per genome); (4) ER2796 *E.coli* gDNA no added nicks; (5) m6A positive control: ER2683 *E. coli* gDNA (Dam^+^) with no enzyme. Lanes 2–4 are in the reverse order as the bars shown in Panel A.

To test the coupling of MTase activity with polymerase activity for 6xHis::Xa::M.EcoGIX::PolI, we used the protein to carry out a nick-translation reaction on unmethylated ER2796 gDNA (Figure 5, Panel A). Nick translation occurs at a DNA break with a 3′OH: PolI polymerizes from the 3′ end while carrying out excision with its 5′ exonuclease, thus moving the nick and transiently exposing the template strand. Genomic DNA as isolated carried some priming sites; adding nicks using site-specific nicking enzymes Nt.CviPII (438,784 CCD sites per genome) and Nt.BspQI (671 GCTCTTC sites per genome) provided additional sites. As expected, polymerization increased with increasing nick density (gDNA<Nt.BspQI<Nt.CviPII; measured with labelled nucleotide, H^3^[dTTP]). Increasing nick density also increased methylation, measured with H^3^[SAM]; furthermore, when nucleotides were added as well as H^3^[SAM] more label as incorporated. Modification of heavily nicked DNA in the presence of nucleotides was 4 times higher than modification of DNA with neither added nicks nor added nucleotides.

A different detection approach used antibody to m6A rather than radioactivity to assess modification level. Nick translation reactions were dot blotted and developed with anti-m6A antibodies (NEB, MA) (Figure 5, Panel B). All DNAs were incubated in the presence of SAM, dNTP and TaqI DNA ligase to seal the nicks. ER2796 *E. coli* DNA as isolated, or treated with endonucleases to produce different densities of nicked non-modified substrate, was detected at higher dilutions with more nicks (Figure 5, Panel B, lanes 4 < 2 < 3). Untreated ER2796 (DNA negative control for dot-blotting) was not recognized by the antibody (Figure 5, Panel B, lane 1); Dam^+^ (G(m6A)TC-modified) *E. coli* DNA from ER2683 provides an indication of sensitivity (Figure 5, Panel B, lane 5).

### Protection of pESBL from recipient restriction depends on *oriT* orientation

Earlier work had shown that M.EcoGIX protected pESBL from restriction by a recipient in a conjugal cross: genetic knockout of the gene *ecoGIXM* encoding the MTase dramatically reduced transfer to strains carrying the EcoGIII restriction system, or to a *K. pneumoniae* isolate with an uncharacterized RM system (43).

**Figure 6.**
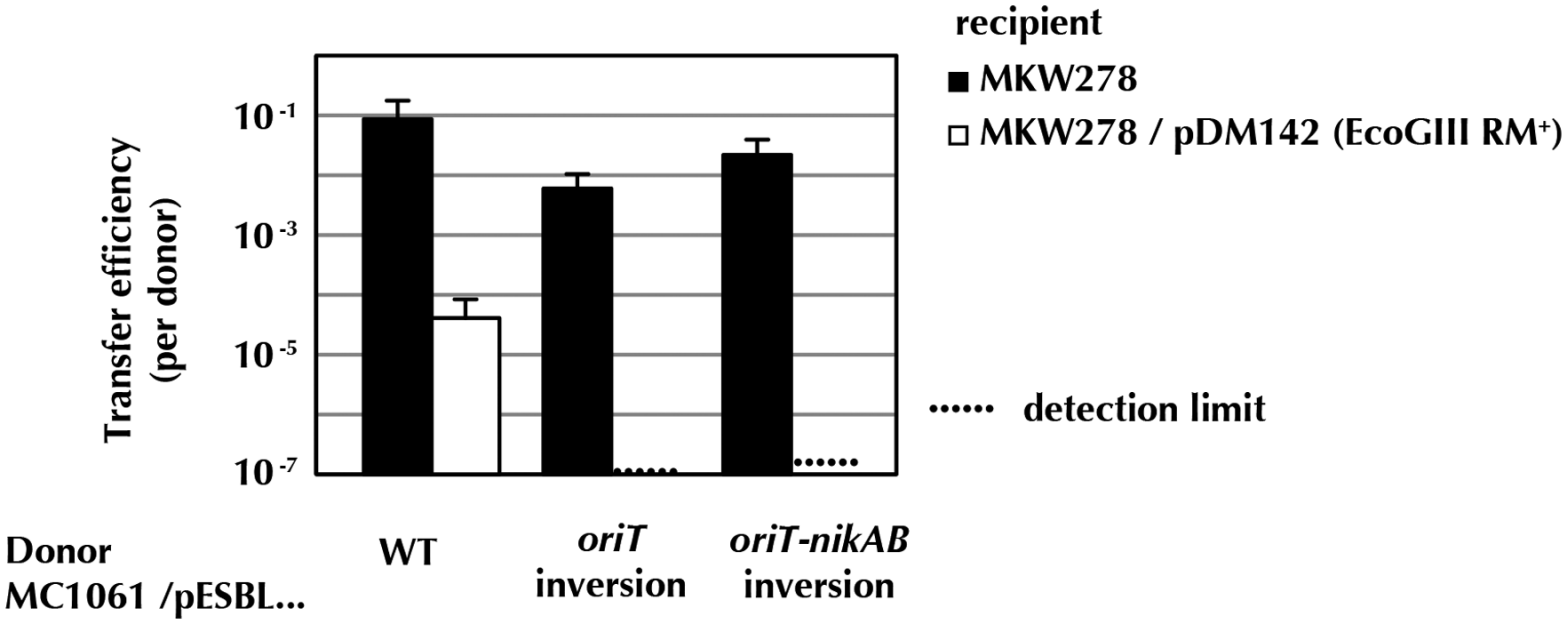
Inversion of pESBL *oriT* in the donor increases sensitivity to EcoGIII restriction in the recipient. When the recipient lacks EcoGIII (MKW278), transfer per donor is mostly unaffected by inversion of the transfer origin (either *ori* or a larger segment with both *ori* and *nikAB*). When the recipient carries the EcoGIII RM system, the wild type plasmid is restricted ∼10^4^-fold; with the inverted origin, transfer is not detected (10^6^–10^7^-fold restriction). EcoGIII does not cleave when the A in CTGC**A**G is modified.

We reasoned that methylation in the donor would still occur regardless of direction of conjugal transfer, but if protection depends on expression of M.EcoGIX in the recipient early after transfer, timing and orientation of transfer of the gene will be critical (28). To distinguish these, we designed two pESBL plasmid variants with inverted *oriT*: pESBL INV (*oriT*) (120bp inverted) and pESBL INV*(oriT-nikAB)* (∼3.8 kb inverted). Mobilization experiments employed laboratory *E. coli* (MC1061 for the donor, MG1655 derivative MKW278 (11) for the recipient) with the EcoGIII RM system present in the recipient or not. As shown in Figure 6, both inversions lead to stronger restriction, of magnitude similar to that previously described for the M.EcoGIX knockout mutant (43). We conclude that the strand transferred to the recipient cells is not modified in the donor. M.EcoGIX activity must be expressed in the recipient.

## DISCUSSION

The role of DNA methyltransferases and more generally the role of epigenetics in the biochemistry and physiology of prokaryotic organisms are actively expanding fields of inquiry, in part due to the proliferation of candidate objects of study. MTases act as genome protectors during RM processes, as has been long-established. However, recent advances in SMRT sequencing has enabled the discovery of numerous MTases not obviously connected with restriction partners. Almost 50% of prokaryotes analyzed harbor ‘orphan’ Type II MTases with no apparent cognate RE (5).

M.EcoGIX and M.BceJIII represent one family of these orphan MTases and are characterized in this work. Unlike many orphan families, they are silent during laboratory growth of the original organisms. Large numbers of M.EcoGIX homologs are found in conjugal plasmids of the IncI1 and IncF families. The IncI1 plasmid pESBL, which specifies M.EcoGIX, has been extensively studied and characterized, due to interest in the pathogenic Shiga toxin that it also carries (42,43). Related proteins are widely distributed in bacteria: BLAST searches identify >1000 potential homologues of the *E. coli* example in GenBank, the vast majority of them plasmid associated (examples can be found in Supplementary Table S7). Understanding the role of the MTases in the dissemination and spread of these plasmids is thus of general interest.

Earlier work had shown transcription of the *ecoGIXM* gene in its native context but neither plasmid nor host were modified at SAY sites (11). Genetic knockout of this MTase gene in this plasmid did dramatically reduce the level of successful plasmid mobilization from the mutated donor to recipients expressing restriction: EcoGIII or a *K. pneumoniae* isolate with uncharacterized R-M systems (43). The lack of donor modification detected by SMRT sequencing was thus puzzling.

We have combined molecular genetic approaches with biochemistry here to enable partial characterization of the enzymes and outline their role in the cell.

### Modification is targeted to the leading strand when borne by *polA*-dependent plasmids

In our artificial expression constructs, constitutive transcription and translation of the MTase genes uncouple their action from the act of conjugation. Plasmid replication in these convenient vectors is unidirectional, initiated by PolI action on the RNAII primer of ColE1 or its counterpart in p15A. We propose that MTase expressed from elsewhere on the plasmid (or from another plasmid) associates with PolI, marking the leading-strand with modification readable by the RSII sequencing protocol (Figure 1). The persistence of the R-loop associated with this replication (55,56) may assist by providing a long-lived single-stranded substrate.

### PolI-dependent plasmids are excluded from genomic competition in the presence of active MTase

Mutations in the presumed catalytic sites (Supplementary Figure S1) have facilitated this analysis. Wild type alleles provide sensitivity to restriction *in vivo* by the modification dependent enzyme EcoKMrr (Supplementary Table S4); our mutations relieve that restriction. Unexpectedly, the MTase wild-type alleles and consequent methylation correlate with a strong depressive effect on plasmid copy number (Table 1), an effect that is relieved by catalytic-site mutants; MTase activity also adds to depression of copy number caused by inactivation of PolI by thermosensitive mutation (Supplementary Figure S3). Two possible explanations occur to us. First, MTase interference could be due to persistent binding of hemimethylated sites in the plasmid origin promoter, *rnaIIp*, by SeqA (57,58). A more general speculative mechanism would invoke the inherent property of methylases to flip the base that is to be modified into a binding pocket. This could lead them to slow down fork progress, leading to lower plasmid copy number.

### Single-strand phage M13 is modified *in vivo* but growth-handicapped

Another possible biological target for a single-strand activity is bacteriophage M13. In Table 2, we find that M13mp18 is modified, but is even more growth-handicapped than are the PolI-dependent replicons. Again, the effect is abrogated by the cataytic-site mutations.

### Single stranded DNA is the substrate *in vitro*

Despite several attempts, the native MTases could not be characterized *in vitro*, but His-tagged versions could be shown to modify single stranded M13mp18 but not the double-stranded form (Figure 2). RNA is not a substrate, either single-stranded or double stranded forms (Supplementary Figure S4; DNA substrates tested in parallel summarized in Supplementary Table S5).

### Modification activity appears in association with DNA Polymerase I

The consistent loss of activity during purification attempts (Supplementary Figure S2) suggested loss of a required factor. Such a factor could be a small molecule or a host protein. Pursuing a protein partner, the fractions through the purification were analysed by LC–MS (Supplementary Table S6).

Since the peculiar behavior of plasmid modification *in vivo* suggested a factor at the replication fork, we chose to pursue PolI as a candidate collaborator. Using the His-tagged version of the MTase, co-immunoprecipitation was used to verify that PolI partners with the MTase by reciprocal immunoprecipitation, Western blotting and enzymatic assays for polymerase and MTase (Figure 3).

### Protein fusion with DNA Polymerase I both modifies and polymerizes

Adding back purified PolI holoenzyme or Klenow fragment did not restore MTase activity to the purified MTase fractions. Quite likely, another host protein plays an intermediary role in matching the two; for example, PolI is known to interact specifically with the sliding clamp at the replication fork (59,60).

A shortcut to enable further characterization was adopted: tether the MTase protein to PolI with an amino acid linker. This was successful (Figure 4). A purifiable MTase activity copurified with Pol activity. This allowed demonstration of modification on single-stranded oligonucleotides but not the double-stranded form of the same oligos (Supplementary Figure S5). The fusion modifies in the absence of polymerization.

Connecting the roles of the two parts of the chimeric protein, Figure 5 shows that both polymerization and modification activities depend on the density of available priming sites, created by site-specific nicking enzymes; modification activity is potentiated by polymerase action.

### A role for M.EcoGIX in protecting the plasmid from restriction on entry in a new host

The PolI dependence and single-strand MTase specificity demonstrated here allows reconciliation of two observations: M.EcoGIX protects conjugal transfer during matings, but no modification is found in the donor. There are two ways to achieve conjugation-coupled modification: the M.EcoGIX MTase might associate with the conjugation assembly in the donor, so that the strand transferred is methylated during transfer. Alternatively, M.EcoGIX might modify the entering single strand in the recipient. The latter requires MTase expression from the single strand, as is known for other anti-defense proteins of conjugal plasmids (see Introduction).

**Figure 7.**
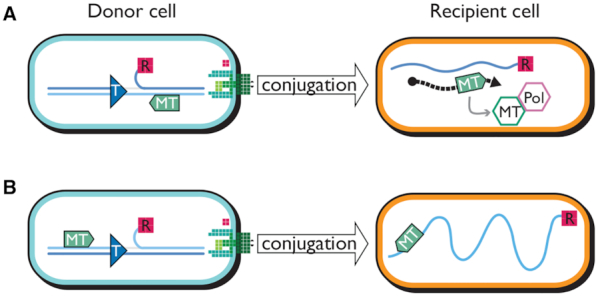
Conjugal DNA transfer positions the MTase gene for expression and coordinated action with PolI in the native orientation but not with inverted *oriT*. Blue lines are DNA; light blue carries the coding sequence for the MTase gene (green arrow); dark blue carries the F*rpo* promoter. (**A**) In the native orientation the relaxase (R) nicks at *oriT* (T), becoming covalently attached to the 5′ side of the nick. Leading strand synthesis from the 3′ side of the nick, and interaction of relaxase with the conjugal apparatus (green structure at the cell periphery), conveys the displaced single strand into the recipient. Transcription (dashed black arrow) from F*rpo* on the conjugal DNA strand allows translation of the MTase gene. This MTase (green hexagon) can then interact with endogenous PolI (lavender hexagon) to modify the single conjugal strand as lagging strand synthesis proceeds from F*rpo* transcripts (not shown). (**B**) When *oriT* is inverted, the MTase gene is transferred late and on the wrong strand for expression, leaving the double-stranded product (not shown) sensitive to restriction.

If the MTase is to be expressed in the recipient, it must be coded for on the entering strand, placed downstream of a promoter that can form from the single strand (Figure 7). Reversal of the orientation of transfer thwarts both requirements (28). As shown in Figure 6, such inversion leads to drastic suppression of transfer into a cell expressing M.EcoGIII.

Note that other anti-defense proteins of pESBL are also implicated by the experiment of Figure 6. In Figure 6 the inversions partially suppress entry even without EcoGIII in the recipient. Recipient MKW278 is one step from MG1655, and likely expresses the Type I enzyme EcoKI. EcoKI restriction can be countered with ArdA (61), a homolog of which is present on pESBL (43).

### A picture of plasmid anti-defense efforts during entry expression

M.EcoGIX and its homologs are found in the early-transferred regions of numerous conjugal plasmids. As described in the introduction, this neighborhood includes many anti-defense factors that thwart cellular regulators of DNA exchange. Once vegetative synthesis has been established, the interests of the transconjugant plasmid are aligned with its (new) host–to forbid entry to other parasitic elements; the early functions thus are silent.

The action of M.EcoGIX at single-stranded regions is consistent with this picture of a role in establishment. By targeting single-stranded regions before or during duplex formation, it provides defense against RE cleavage of the duplex, while remaining inactive during normal cell behavior. The low level of modification is also consistent with the observation that over-methylation can prove lethal as is observed for the non-specific MTase, M.EcoGII (62)—a prophage-encoded MTase that is also silent except during phage development. The degenerate recognition site SAY is consistent with RE defense, since very many different RE specificities are likely to be encountered during horizontal transfer,.

The PolI association of M.EcoGIX is also consistent with this early role in establishment. While lagging-strand synthesis is forming the duplex, the primer-digestion activity of PolI will target the MTase to its template. Once the secondary structure of the F*rpo* (*ssi*) upstream of *ecoGIXM* is ironed out into duplex DNA, transcription of the MTase will cease, eliminating the physiologic disruption likely to be engendered by extensive modification of the transconjugant's cellular promoters. Inversion of the direction of transfer, as in Figure 7, results in placement of *ecoGIXM* on the wrong strand to allow expression.

Since the 5′-nuclease of PolI is essential for replication to excise Okazaki fragments, one might have expected to see more methylation of the lagging strand. Some is observed, but mostly at very low levels. Perhaps a low-level activity reflects the limited time that the PolI-M.EcoGIX complex actually spends on the lagging strand. This combined with its sequence dependence may explain the observed asymmetry.

## Supplementary Material

gkaa1163_Supplemental_File
