# Supplementary material for: Plasmid replication-associated single-strand-specific methyltransferases
Source: Nucleic Acids Res. 2020 Dec 3;48(22):12858–73. doi: 10.1093/nar/gkaa1163 (PMC7736820; doi:10.1093/nar/gkaa1163)
Supplement: gkaa1163_Supplemental_File [file gkaa1163_supplemental_file.pdf]

SUPPLEMENTARY TABLES AND FIGURES.

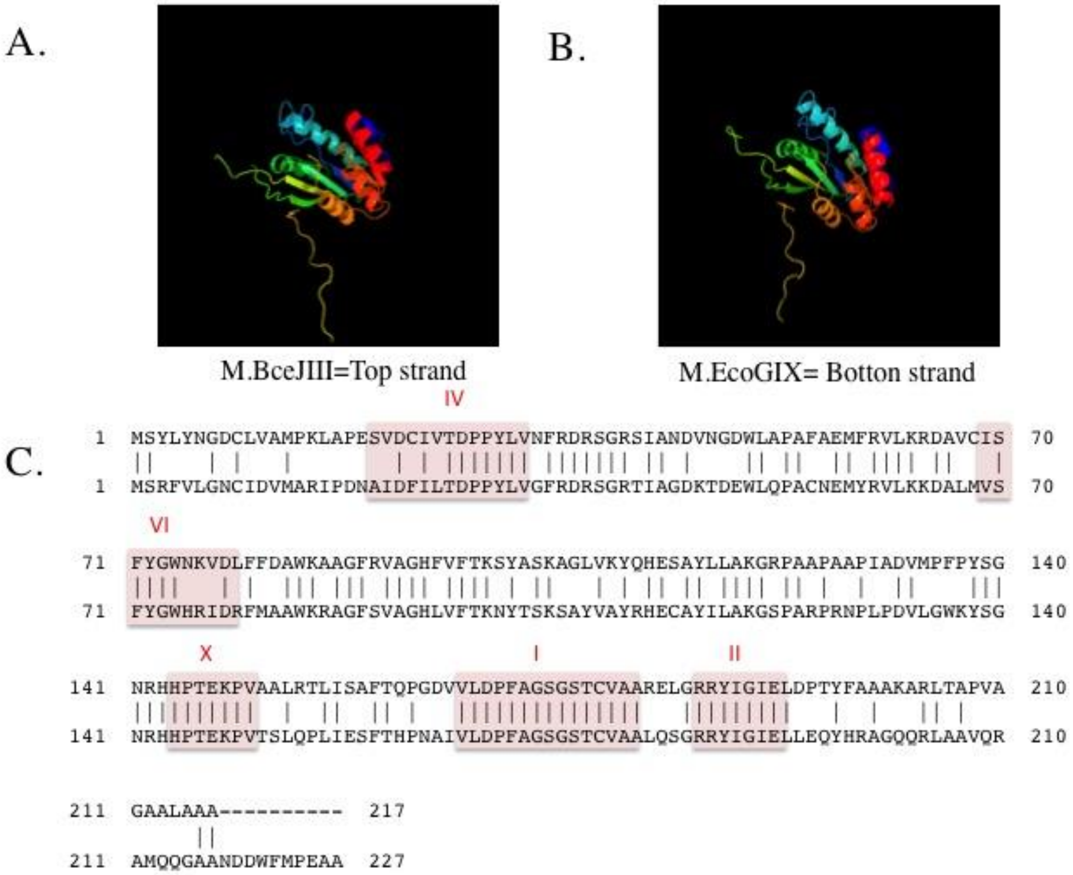

Supplementary Figure 1. Structural modeling analysis of A) M.BceJIII and B) M.EcoGIX using Phyre2 (**P**rotein **H**omology/analog**Y** **R**ecognition **E**ngine V2.0). C) Amino-acid sequence alignment of M.BceJIII and M.EcoGIX. Roman numerals correspond to conserved motifs in m6A DNA MTases.

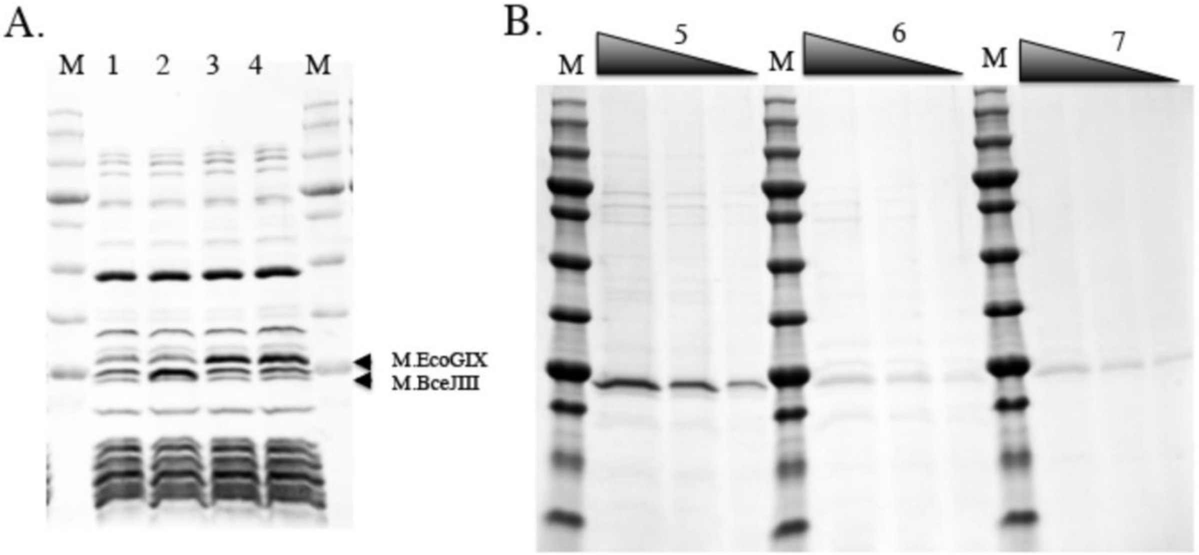

	PureExpress				T7 express *E.coli*		
	1	2	3	4	5	6	7
Construct	Vector	M.BceJIII wt	M.EcoGIX wt	M.EcoGIX mut	M.BceJIII wt	M.EcoGIX wt	M.EcoGIX mut
Crude extract H^3 DNA cpm	22	5067	68639	322	17584	300153	340
Purified proteins H^3 DNA cpm					65	34	31

Supplementary Figure 2. Activity loss during purification of His-tagged variant proteins. Panel A: PURExpress reactions from 1) empty vector pSAPv6 or 6xHis-tagged Xa linked variants of 2) pAF9: M.BceJIII WT, 3) pAF10: M.EcoGIX WT and 4) pAF11: M.EcoGIX APPA mutant. Panel B: Dilutions fractions from three-column purified fractions from lysates of T7 Express (ER2566) expressing 5) M.BceJIII WT, 6) M.EcoGIX WT and 7) M.EcoGIX APPA variant. Panel C: MTase activity measured with [H^3]SAM with single-stranded M13mp18 DNA as a substrate. Top row: lanes 1-4, activity in the PureExpress reactions of panel A; lanes 5-7 activity measured in crude extracts of the expression constructs. Bottom row: activity measured following purification (fractions of panel B).

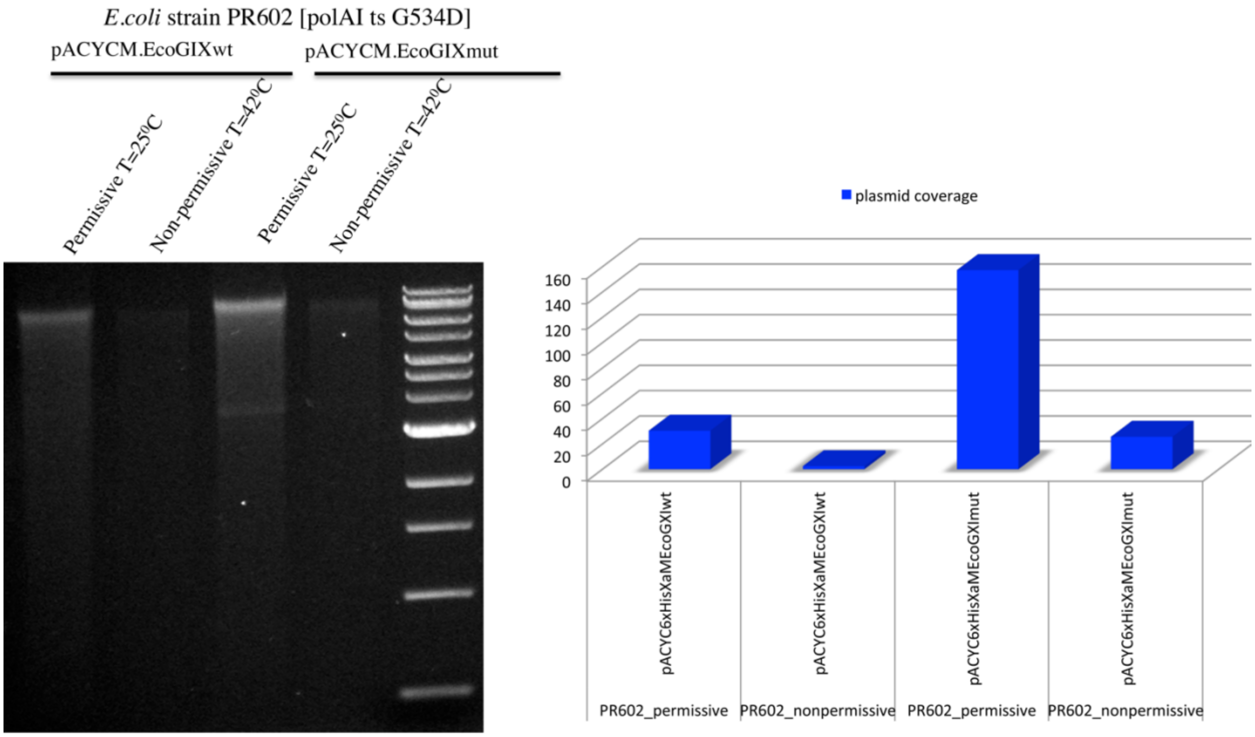

Strain	PR602_permissive	PR602_non-permissive	PR602_permissive	PR602_non-permissive
**plasmid**	pACYC6xHisXaMEcoGXIwt	pACYC6xHisXaMEcoGXIwt	pACYC6xHisXaMEcoGXImut	pACYC6xHisXaMEcoGXImut
**plasmid coverage**	30.46972477	2.390169456	157.3182333	25.65491252

Supplementary Figure 3. p15A copy number depends on both plasmid *ecoGIXM* allele and chromosomal *polA*. Ethidium bromide-stained agarose gel of plasmid DNA carrying WT or the APPA mutant of *ecoGIXM* isolated from *polAts* cells grown at the permissive or nonpermissive temperature. Sequencing yielded estimates of copy number per chromosome as in Figure 2. Lane 1 and 2: pAF5, *ecoGIXM* WT; Lanes 3 and 4 pAF6, *ecoGIXM* mut

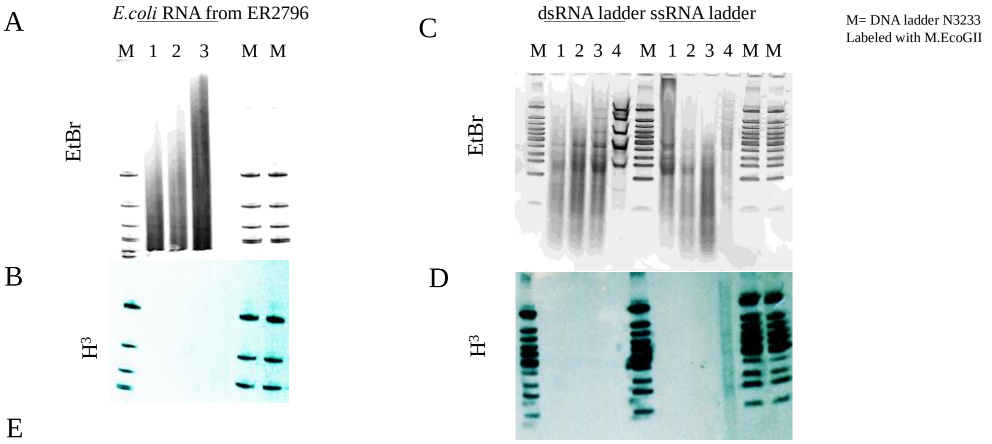

E

		H3 cpm							
	Samples	E.coli RNA	E.coli RNA +DNaseI	ds RNA ladder (N0363)	ss RNA ladder (N0362)	DNA ladder (N3233)			
	Total	4,478,600	4,478,600	4,478,600	4,478,600	4,478,600			
1	PureExpress pSAPv6	23	27	13	11				
2	PureExpress M.BceJIII (pAF9)	29	35	13	15				
3	PureExpress M.EcoGIX (pAF10)	615	38	46	180				
4	M.EcoGII	2,387	1,842	172	1,443	51,435			

Supplementary Figure 4 RNA is not a substrate.
[H^3]SAM labelling reactions: Panels A and B, substrate was total RNA from a non-modifying host; panels C and D, substrates were double-stranded (N0363) and single-stranded RNA (N0362) size markers. Substrates (1 µg) incubated with 5 µl of PurExpress products of Lanes 1: Vector pSAPv6; lanes 2 pAF9 expressing M.BceJIII; lanes 3: pAF10 expressing M.EcoGIX; or with lanes 4: control DNA MTase M.EcoGII. Control lanes M: DNA ladder N3233 substrate labelled with nonspecific M.EcoGII. Panels A and B: photographs of EtBr-stained gels; Panels B and D: Fluorographs of the gels of A and B. Panel E: counts of total [H^3] in each reaction before and after repurification of RNA with Monarch RNA purification columns. The same enzyme sources were used to modify single- and double-stranded M13 DNA in Supplementary Table 5.

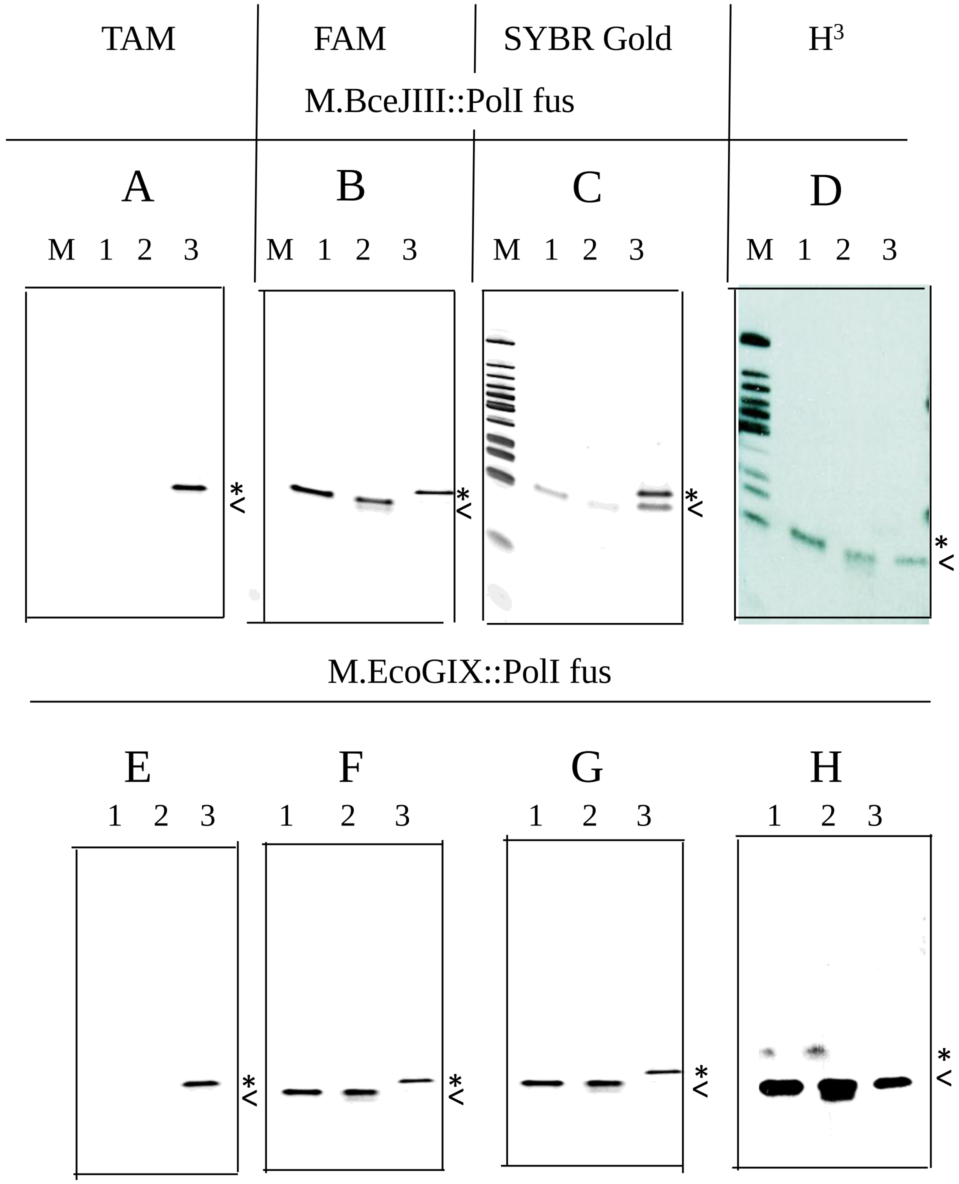

Supplementary Figure 5. Single- but not double-stranded oligonucleotides are modified by PolI-MTase fusions.

Complementary oligonucleotides labelled with TAM (lanes 1; #464 of Supplementary Table 3) or FAM (lanes 2; #467 of Supplementary Table 3) were annealed (lanes 3) and used as substrates for modification reactions in the presence of [H^3]SAM. M: NEB N3233 marker labeled at A residues with [H3]SAM. (*): the position of the annealed (ds) oligonucleotides; (<): position of the single-stranded oligonucleotide. MTase reactions were active (QHP) methylase fractions of fusion proteins 6xHis::M.BceJIII::PolI (panels A-D) and 6xHis::Xa:M.EcoGIX::PolI (panel E-H). A Typhoon reader detected TAM (488nm), FAM (532nm) and SYBR Gold (detecting DNA). [H^3]-modified DNA (Panels D and H) was detected by fluorography as described in Materials and Methods. The annealed species (lanes 3) still contains some single-stranded substrate, most visible in panel C. The FAM-labelled oligo (lanes 2) shows a small amount of a faster-moving species, most visible in panel H. More-pronounced labelling by M.EcoGIX::PolI than M.BceJIII::PolI is consistent with modification activity seen in Figure 4.

Supplementary Table 1. List of Strains.

E. coli strains	Genotype/Phenotype	Source	Reference
ER2683	Cloning strain: F128 (ER2250) *proA+B+ lacIq Δ(lacZ)M15 zzf:: miniTn10(KanR)/ fhuA2::IS2 Δ(lacI-lacA)200 glnX44 e14- rfbD1? relA1? endA1 spoT1? thi-1 Δ(yjiT-opgB)114::IS10*	Raleigh E. (NEB)	Lin,L.F., Posfai,J., Roberts,R.J. and Kong,H. (2001) Comparative genomics of the restriction-modification systems in Helicobacter pylori. Proc Natl Acad Sci USA, 98, 2740-2745.
ER3081	T7 expression strain: *IN(IS1_26-Δ(yjiT-opgB)114::IS10-IS1_1) fhuA2::IS2 lacZ::T7gene1 [plon::IS186] del[aaad-ompT-cusS] del(galK-yghJ) psulA211(-37 T->C) uvrY::IS1::del(yecF-dcm-hchA) mcrX73::(mTn10 tetA::IS10) IN(speA210::IS10-xanA::IS10) endA1(E208K) attB::(pCD13-lysY, lacIq)*	Samuelson J. (NEB)	Platt,R., Drescher,C., Park,S.-K. and Phillips,G. (2000) Genetic system for reversible integration of DNA constructs and lacZ gene fusions into the Escherichia coli chromosome. Plasmid, 43, 12–23.
ER2796	Methylation-deficient strain: *fhuA2 Δ (lacZ)r1 glnV44 trp-31 dcm-6 his-1 zed-501::Tn10 argG6 rpsL104 dam-16::Kan xyl-7 mtl-2 metR1 mcr-62 Δ (mcrB-hsd-mrr)114*	Anton B., et al. (NEB)	Anton B.P., Mongodin E.F., Agrawal S., Fomenkov A., Byrd D.R., Roberts R.J,. Raleigh E.A. (2015) Complete Genome Sequence of ER2796, a DNA Methyltransferase-Deficient Strain of Escherichia coli K-12. PloS One, 10 (5)
ER3661	Methylation-deficient strain to propagate M13 phage: F'128 *proABlacIq 'ZΔM15 mini-Tn10Cm/ fhuA2 Δ (lacZ)r1 glnV44 trp-31 dcm-6 his-1 zed-501::Tn10 argG6 rpsL104 dam-16::Kan xyl-7 mtl-2 metR1 mcr-62 Δ (mcrB-hsd-mrr)114*	This study	
MKW278	MG1655 *ΔlacZ::cat*	Yamaichi 2015	Yamaichi, Y., Chao, M.C., Sasabe, J., Clark, L., Davis, B.M., Yamamoto, N., Mori, H., Kurokawa, K., Waldor, M.K. (2015) High-resolution genetic analysis of the requirements for horizontal transmission of the ESBL plasmid from Escherichia coli O104:H4. Nucleic Acids Res., 43, 348-360.
YBB1214	MKW278 / pDM142 (pBAD33 EcoGIIIr+m+)	Yamaichi 2015	
bEYY1132	SM10 lambda pir / pEYY56	This study	
bEYY1133	SM10 lambda pir / pEYY57	This study	
bEYY1137	MC1061 / pESBL INV (*oriT*)	This study	
bEYY1138	MC1061 / pESBL INV (*oriT-nikAB*)	This study	

Supplementary Table 2. List of Plasmids.

Supplementary Table 2 Plasmids.xlsx

**Plasmids and Phage DNA**				**Expression promot**
**Plasmid name**	**in process name**	**Origin**	**promoter**	**Mtase**
pRRS	pRRSlac	ColE1	*lacp*	(vector)
pAF1	pRRStetM.BceJIII wt	ColE1	*tetp*	BceJIII
pAF2	pRRStetM.BceJIII mut	ColE1	*tetp*	BceJIII
pEYY63	pEYY63	ColE1	*lacp*	EcoGIX
pAF3	pRRSlacM.EcoGIX wt	ColE1	*lacp*	EcoGIX
pAF4	pRRSlacM.EcoGIX mut	ColE1	*lacp*	EcoGIX
pAF5	pACYCDtet_6xHis::Xa::M.EcoGIXwt	p15A	*tetp*	EcoGIX
pAF6	pACYCDtet_6xHis::Xa::M.EcoGIXmut	p15A	*tetp*	EcoGIX
pAF7	pSYX20_Cm_6xHis::Xa::M.EcoGIXwt#2	pSC101	*tetp*	EcoGIX
pAF8	pSYX20_Cm_6xHis::Xa::M.EcoGIXwt#5	pSC101 inv	*tetp*	EcoGIX
pAF9	pSAPV6xHis::MBceJIIIwt	p15A	*T7p*	BceJIII
pAF10	pSAPV6xHis::Xa::M.EcoGIX wt	p15A	*T7p*	EcoGIX
pAF11	pSAPV6xHis::Xa::M.EcoGIX mut	p15A	*T7p*	EcoGIX

Supplementary Table 3. List of Oligonucleotides and primers.

PCR and cloning oligos

Accession #	In process name	sequence
P1	pRRStet_M.BceJIII_Sbf_NdeIF _69	ttagttgCCTGCAGGttaaggttaacatATGAGCTATTTGTACAACGGCGACTG
P2	pRRStet_M.BceJIII_NdeI_His_ Xa_F	tttatactgcagcatATGcaccatcaccatcaccatATAGAGGGGCGGAGCTATTTGTAC AACGGCGACTG
P3	pRRStet_M.BceJIII_BglIIR	CGCAGATCTTTAGGCGGCGGCCAACG
P4	pM.BceJIII_72F_mut	TTTTCGCGATCGCAGCGGC
P5	pM.BceJIII_APPA_R_mut	TTCACGAGGgcAGGCGGGgCCGT
P6	pRRSlac_M.EcoGXI_PstI_Nde IF	ttagttgCTGCAGttaaggttaacatATGTCCCGTTTTGTCCTCGGTAAC
P7	pRRSlac_M.EcoGXI_BamHR	cattcaggatccTTATTACGCAGCCTCCGGCATAAAC
P8	pACYC_6xHis_Xa_MEcoG_N de_67F	caggaaggtttaacatATGcaccatcaccatcaccatATAGAGGGGCGGTCCCGTTTTGT CCTCGGTAAC
P9	pACYC_M.EcoGIX_Bam_69R	gaggtgccgccggcttccattcaggatccTTATTACGCAGCCTCCGGCATAAAC
P10	pMEcoGIX_73F_mut	GTTTTCGTGACCGTTCCGG
P11	pMEcoGIX_APPA_R_mut	CGACGAGGgcCGGCGGggcAG
P12	pACYC_tet_64_vector_rev	catatgttaaaccttcCTGCGTTAGCAATTTAACTGTGATAAACT
P13	pACYC_tet_64_vector_for	taaGGATCCtgaatggaagccggcg
P14	oYo157	ATGTTCTTTCGAAAACTCACGTTAAGGGATTTTGGT
P15	oYo158	TCAGTGGAACCTGCGTTATCCCCTGATTCT
P16	oYo159	GTGAGTTTTCGAAAGAACATGTGAGCAAAAGGCCAGC
P17	oYo160	GATAACGCAGGTTCCACTGAGCGTCAGACCC
P23	oYo107	GCGGAGTGTATATCAAGCTTATCGAAAGCCCTGGTATTTATGCC
P29	oYo109	GAATGTACTGAACAGTGGCCGTAGTCTTATGCAGACGGCAG
P28	oYo111	TTGTGAGCGGATAACAATTTGTGGTTGACCGCAACGTGAACATG
P30	oYo112	CTGCCGTCTGCATAAGACTACGGCCACTGTTCAGTACATTC

P27	oYo142	GCCTGTTATTTTTGTGCATCGTAAGGAGCCTGAAGTGAGTG
P32	oYo143	GGCCGCGCCAGAATCCGGCGTAGTCTTATGCAGACGGCAG
P34	oYo144	CTGCCGTCTGCATAAGACTACGCCGGATTCTGGCGCG
P35	oYo145	GAATGTACTGAACAGTGGCCGTGATGCACAAAAATAACAGGC
P33	oYo146	GCCTGTTATTTTTGTGCATCACGGCCACTGTTCAGTACATTC
*same as P14	oYo157	ATGTTCTTTCGAAAACTCACGTTAAGGGATTTTGGT
*same as P15	oYo158	TCAGTGGAACCTGCGTTATCCCCTGATTCT
*same as P16	oYo159	GTGAGTTTTCGAAAGAACATGTGAGCAAAAGGCCAGC
*same as P17	oYo160	GATAACGCAGGTTCCACTGAGCGTCAGACCC
	pSAPv6_6xHis_69F	tttaagaaggagatatacatATGcaccatcaccatcA
P19	pSAPV6_PolAI_BamH_67R	gggtcgacgaagagcggatccTTAGTGCGCCTGATCCCAG
P20	PolAI_Spe_67F	TTCAATTTGTAATACCTAATCGTGGTGTGACTAAGCAactagtcttgATG GTTCAGATCCCCCAAAATCC
P21	pEcoGIX_WO_stop_Nhe_69R	GATTAGGTATTACAAATTGAAGAATATGGTCTTTgctagcATACGCAG CCTCCGGCATAAAC
P22	pBceJIII_WO_stop_Nhe_69R	GATTAGGTATTACAAATTGAAGAATATGGTCTTTgctagcATAGGCG GCGGCCAAC

PacBio adapter

	PacBio adapter_direct	5'P-ATCTCTCTCTTTTCCTCCTCCTCCGTTGTTGTTGTTGAGAGAGAT
	PacBio adapter_rev_complement	5'P- ATCTCTCTCAACAACAACAACGGAGGAGGAGGAAAAGAGAGAG AT

Sequencing primers

	pTetF	GTTGTAATTCTCATGTTTGACAGC
	pTetR	GTTCTGCCAAGGGTTGGTTTGCGC
	S1224	CGCCAGGGTTTTCCCAGTCACGAC
	S1233	AGCGGATAACAATTTCACACAGGA
	S1248	TAATACGACTCACTATAGGG
	S1271	TATGCTAGTTATTGCTCAG

M.BceJIII Modification substrate

464_TAM	two SAY sites indicated in bold and underlined	5'TAM-GTATA**GAT**GT**GAT**GAATATACCTGTTTATGTGGCAACCGAAATAGTTATG-464_TAM
467_FAM	eight SAY sites indicated in bold and underlined)	5' FAM-TG**GACAT**AACTATTTCGGTTGC**CACAT**AAACAGGTATATT**CATCACAT**CT-467_FAM

Supplementary Table 4. Active MTase alleles confer sensitivity to EcoKMrr.

			Recipient host:	Permissive (Mrr^+)		Restrictive (Mrr^-)		R/P
**Plasmids (**from ER2796)								
	Replicon	Allele	Promoter	Col per tf	Morph	Col per tf	Morph	~EOT
pAF5	p15A	M.EcoGIX wt	*tetp*	$\sim10^5$	S (17 N)	5	N*	~1E-4
pAF6	p15A	M.EcoGIX mut	*tetp*	$\sim10^5$	N	$\sim10^5$	N	~1
pAF3	ColE1	M.EcoGIX wt	*lacp*	$\sim10^5$	N	$\sim10^3$	S	~0.01
pAF4	ColE1	M.EcoGIX mut	*lacp*	$\sim10^5$	N	$\sim10^5$	N	~1
pAF1	ColE1	M.BceJIII wt	*tetp*	$\sim10^6$	N	$\sim10^3$	S	~0.001
pAF2	ColE1	M.BceJIII mut	*tetp*	$\sim10^6$	N	$\sim10^5$	N	~0.1
pAF7	pSC101	M.EcoGIX wt	*tetp*	$\sim10^6$	N	$\sim10^6$	N	~1

Colonies per transformation reaction (Col per tf) upon transformation (10 ng of plasmid) into permissive (ER1969, Mrr^-) or restrictive (ER1516, Mrr^+) strains when plated selectively. Morph (morphology): S, small: <0.5 mm; N, normal, ~2 mm after overnight incubation; EOT: efficiency of transformation (restrictive host (R)/permissive host (P)). The propagation host ER2796 does not modify, so only plasmid-specified modification is present. Plasmids carried expression configurations of wild type (WT) or disabled (mut) alleles of *ecoGIXM* (Sm6AY) or *bceJIIIM* (m6A). *chose for sequencing

Supplementary Table 5. DNA substrate control for Supplementary Figure 4

		H3 cpm		
	**Samples**	**M13mp18 SS DNA**	**M13m18 RFI**	**lambda HindIII**
	Total cpm added	4,478,600	4,478,600	4,478,600
1	PureExpress pSAPv6	24	50	
2	PureExpress M.BceJIII (pAF9)	601	24	
3	PureExpress M.EcoGIX (pAF10)	15,338	622	
4	M.EcoGII	205,370	135,510	144,700
5	Pure Express M.EcoGIX::PolI (pAF14)	8,357	153	

Supplementary Table 6. Summary of polypeptides interacting with active and inactive 6xHis::M.BceJIII detected by LC-MS.

Control #Spec	Active MBceJIII_QFF #Spec	Inactive MBceJIII_QFF _Hep #Spec	Avg. Mass	Description
0	51	247	24819	6xHisX_M.BceJIIIwt
3	87	5	103118	gb ACT45542.1 DNA polymerase I [Escherichia coli BL21(DE3)]
0	57	45	40324	gb ACT41997.1 cell division protein FtsZ [Escherichia coli BL21(DE3)]
9	481	9	17545	gb ACT44958.1 30S ribosomal protein S5 [Escherichia coli BL21(DE3)]
0	370	0	22244	gb ACT44975.1 50S ribosomal protein L3 [Escherichia coli BL21(DE3)]
0	66	0	70546	BL21(DE3)]
0	80	1	78526	gb ACT45071.1 4-alpha-glucanotransferase (amylomaltase) [Escherichia coli BL21(DE3)]
0	122	0	9684	BL21(DE3)]
0	206	0	11316	BL21(DE3)]
4	309	10	17712	BL21(DE3)]
2	235	9	37973	gb ACT44368.1 recombinase A [Escherichia coli BL21(DE3)]
0	79	0	92109	BL21(DE3)]
3	276	0	14966	BL21(DE3)]
0	529	6	9535	gb ACT45669.1 HU DNA-binding transcriptional regulator alpha subunit [Escherichia coli BL21(DE3)]
2	64	1	21074	gb ACT46031.1 periplasmic protein [Escherichia coli BL21(DE3)]
0	106	0	6372	BL21(DE3)]
6	26	6	49594	gb ACT45609.1 ATP-dependent protease ATP-binding subunit [Escherichia coli BL21(DE3)]
5	47	1	35048	gb ACT43641.1 fused mannose-specific PTS enzymes: IIA component/IIB component [Escherichia coli BL21(DE3)]

Supplementary Table 7. List of M.EcoGIX homologs

Summary of BLAST against M.EcoGIX was shown (cut-off at E-value <1.00E-50). BLAST program was run at Kyoto Encyclopedia of Genes and Genom
M.EcoGIX homologs encoded on plasmid are shown in black and those encoded on chromosome are shown in red.

Org code*	Organism	bits	E value	coding replicon	coding region
esl	Escherichia coli O104 H4 2011C-3493	478	7.00E-170	pESBL-EA11	54782..55465
elu	Escherichia coli UM146	461	2.00E-163	pUM146	complement(51505..52188)
ese	Escherichia coli O150:H5 SE15 (commensal)	461	2.00E-163	pECSF1	113742..114425
eum	Escherichia coli O17:K52:H18 UMN026 (ExPEC)	461	2.00E-163	p1ESCUM	complement(105909..106592)
eci	Escherichia coli O18:K1:H7 UTI89 (UPEC)	461	2.00E-163	pUTI89	62243..62926
ecy	Escherichia coli O152:H28 SE11 (commensal)	459	1.00E-162	pSE11-1	33937..34620
ecoj	Escherichia coli JJ1886	458	3.00E-162	pJJ1886_5	12491..13174
ell	Escherichia coli W	458	3.00E-162	pRK1	91798..92481
elw	Escherichia coli W	458	3.00E-162	pRK1	98814..99497
eko	Escherichia coli KO11FL	458	3.00E-162	pEKO1101	15429..16112
ecy	Escherichia coli O152:H28 SE11 (commensal)	457	5.00E-162	pSE11-2	24170..24853
eun	Escherichia coli UMNK88	457	1.00E-161	pUMNK88_91	18267..18950
sfe	Shigella flexneri 2002017 (serotype Fxv)	457	1.00E-161	pSFxv_1	163189..163872

Supplementary Table 7. List of M.EcoGIX homologs

Summary of BLAST against M.EcoGIX was shown (cut-off at E-value <1.00E-50).es (KEGG) and their organism code*, along
M.EcoGIX homologs encoded on plasmid are shown in black and those encode

Org code*	Organism	homology
esl	Escherichia coli O104 H4 2011C-3493	Identities = 227/227 (100%), Positives = 227/227 (100%), Gaps = 0/227 (0%)
elu	Escherichia coli UM146	Identities = 219/227 (96%), Positives = 223/227 (98%), Gaps = 0/227 (0%)
ese	Escherichia coli O150:H5 SE15 (commensal)	Identities = 219/227 (96%), Positives = 223/227 (98%), Gaps = 0/227 (0%)
eum	Escherichia coli O17:K52:H18 UMN026 (ExPEC)	Identities = 219/227 (96%), Positives = 223/227 (98%), Gaps = 0/227 (0%)
eci	Escherichia coli O18:K1:H7 UTI89 (UPEC)	Identities = 219/227 (96%), Positives = 223/227 (98%), Gaps = 0/227 (0%)
ecy	Escherichia coli O152:H28 SE11 (commensal)	Identities = 217/227 (96%), Positives = 221/227 (97%), Gaps = 0/227 (0%)
ecoj	Escherichia coli JJ1886	Identities = 216/227 (95%), Positives = 220/227 (97%), Gaps = 0/227 (0%)
ell	Escherichia coli W	Identities = 216/227 (95%), Positives = 221/227 (97%), Gaps = 0/227 (0%)
elw	Escherichia coli W	Identities = 216/227 (95%), Positives = 221/227 (97%), Gaps = 0/227 (0%)
eko	Escherichia coli KO11FL	Identities = 216/227 (95%), Positives = 221/227 (97%), Gaps = 0/227 (0%)
ecy	Escherichia coli O152:H28 SE11 (commensal)	Identities = 216/227 (95%), Positives = 221/227 (97%), Gaps = 0/227 (0%)
eun	Escherichia coli UMNK88	Identities = 215/227 (95%), Positives = 221/227 (97%), Gaps = 0/227 (0%)
sfe	Shigella flexneri 2002017 (serotype Fxv)	Identities = 215/227 (95%), Positives = 220/227 (97%), Gaps = 0/227 (0%)

Supplementary Table 7. List of M.EcoGIX homologs

Summary of BLAST against M.EcoGIX was shown (cut-off at E-value <1.00E-50). BLAST program was run at Kyoto Encyclopedia of Genes and Genom

M.EcoGIX homologs encoded on plasmid are shown in black and those encoded on chromosome are shown in red.

Org code*	Organism	bits	E value	coding replicon	coding region
sfl	Shigella flexneri 301 (serotype 2a)	457	1.00E-161	pCP301	164527..165210
seeh	Salmonella enterica subsp. enterica serovar Heidelberg 41578	457	1.00E-161	pSEEH1578_01	complement(48350..49033)
senh	Salmonella enterica subsp. enterica Serovar Heidelberg CFSAN0020	457	1.00E-161	pCFSAN002069_01	58902..59585
seb	Salmonella enterica subsp. enterica serovar Typhimurium ST4/74	457	1.00E-161	TY474p2	18806..19489
sey	Salmonella enterica subsp. enterica serovar Typhimurium SL1344	457	1.00E-161	pCol1B9_SL1344	18806..19489
ecw	Escherichia coli E24377A (ETEC)	456	2.00E-161	pETEC_73	complement(5475..6158)
seh	Salmonella enterica subsp. enterica serovar Heidelberg SL476	456	3.00E-161	pSL476_91	23880..24563
ecoo	Escherichia coli O145:H28 RM13514 (EHEC)	454	1.00E-160	pO145-13514	21019..21702
eoj	Escherichia coli O26:H11 11368 (EHEC)	454	1.00E-160	pO26_1	54551..55234
sec	Salmonella enterica subsp. enterica serovar Choleraesuis	451	3.00E-159	pSC138	complement(96052..96735)
ecw	Escherichia coli E24377A (ETEC)	443	3.00E-156	pETEC_35	complement(18014..18697)
elh	Escherichia coli O78:H11:K80 H10407 (ETEC)	441	1.00E-155	p666	complement(42959..43642)
elx	Escherichia coli O157:H7 Xuzhou21 (EHEC)	441	2.00E-155	pO157	39364..40047

Supplementary Table 7. List of M.EcoGIX homologs

Summary of BLAST against M.EcoGIX was shown (cut-off at E-value <1.00E-50).es (KEGG) and their organism code*, along
M.EcoGIX homologs encoded on plasmid are shown in black and those encode

Org code*	Organism	homology
sfl	Shigella flexneri 301 (serotype 2a)	Identities = 215/227 (95%), Positives = 220/227 (97%), Gaps = 0/227 (0%)
seeh	Salmonella enterica subsp. enterica serovar Heidelberg 41578	Identities = 215/227 (95%), Positives = 221/227 (97%), Gaps = 0/227 (0%)
senh	Salmonella enterica subsp. enterica Serovar Heidelberg CFSAN0020	Identities = 215/227 (95%), Positives = 221/227 (97%), Gaps = 0/227 (0%)
seb	Salmonella enterica subsp. enterica serovar Typhimurium ST4/74	Identities = 215/227 (95%), Positives = 221/227 (97%), Gaps = 0/227 (0%)
sey	Salmonella enterica subsp. enterica serovar Typhimurium SL1344	Identities = 215/227 (95%), Positives = 221/227 (97%), Gaps = 0/227 (0%)
ecw	Escherichia coli E24377A (ETEC)	Identities = 215/227 (95%), Positives = 220/227 (97%), Gaps = 0/227 (0%)
seh	Salmonella enterica subsp. enterica serovar Heidelberg SL476	Identities = 214/227 (94%), Positives = 219/227 (96%), Gaps = 0/227 (0%)
ecoo	Escherichia coli O145:H28 RM13514 (EHEC)	Identities = 217/227 (96%), Positives = 220/227 (97%), Gaps = 0/227 (0%)
eoj	Escherichia coli O26:H11 11368 (EHEC)	Identities = 217/227 (96%), Positives = 220/227 (97%), Gaps = 0/227 (0%)
sec	Salmonella enterica subsp. enterica serovar Choleraesuis	Identities = 212/227 (93%), Positives = 218/227 (96%), Gaps = 0/227 (0%)
ecw	Escherichia coli E24377A (ETEC)	Identities = 207/227 (91%), Positives = 216/227 (95%), Gaps = 0/227 (0%)
elh	Escherichia coli O78:H11:K80 H10407 (ETEC)	Identities = 208/227 (92%), Positives = 216/227 (95%), Gaps = 0/227 (0%)
elx	Escherichia coli O157:H7 Xuzhou21 (EHEC)	Identities = 206/227 (91%), Positives = 214/227 (94%), Gaps = 0/227 (0%)

Supplementary Table 7. List of M.EcoGIX homologs

Summary of BLAST against M.EcoGIX was shown (cut-off at E-value <1.00E-50). BLAST program was run at Kyoto Encyclopedia of Genes and Genom

M.EcoGIX homologs encoded on plasmid are shown in black and those encoded on chromosome are shown in red.

Org code*	Organism	bits	E value	coding replicon	coding region
etw	Escherichia coli O157:H7 TW14359 (EHEC)	441	2.00E-155	pO157	38985..39668
ecf	Escherichia coli O157:H7 EC4115 (EHEC)	441	2.00E-155	pO157	22903..23586
ecs	Escherichia coli O157:H7 Sakai (EHEC)	441	2.00E-155	pO157	39363..40046
ece	Escherichia coli O157:H7 EDL933 (EHEC)	441	2.00E-155	pO157	61938..62621
elo	Escherichia coli O44:H18 042 (EAEC)	440	3.00E-155	pAA	complement(98577..99260)
eun	Escherichia coli UMNK88	438	3.00E-154	pUMNK88_Hly	62587..63270
setu	Salmonella enterica subsp. enterica serovar Typhimurium U288	438	3.00E-154	pSTU288-1	complement(11966..12649)
elr	Escherichia coli O55:H7 RM12579 (EPEC)	434	6.00E-153	p12579_2	63539..64222
eok	Escherichia coli O55:H7 CB9615 (EPEC)	434	6.00E-153	pO55	63573..64256
eoj	Escherichia coli O26:H11 11368 (EHEC)	433	2.00E-152	pO26_2	8479..9162
seeb	Salmonella enterica subsp. enterica serovar Bareilly	413	2.00E-144	unnamed	complement(49888..50565)
send	Salmonella enterica subsp. enterica serovar Typhimurium DT104	410	3.00E-143	pDT104	49478..50158
setu	Salmonella enterica subsp. enterica serovar Typhimurium U288	410	3.00E-143	pSTU288-1	complement(86987..87667)

Supplementary Table 7. List of M.EcoGIX homologs

Summary of BLAST against M.EcoGIX was shown (cut-off at E-value <1.00E-50).es (KEGG) and their organism code*, along

M.EcoGIX homologs encoded on plasmid are shown in black and those encode

Org code*	Organism	homology
etw	Escherichia coli O157:H7 TW14359 (EHEC)	Identities = 206/227 (91%), Positives = 214/227 (94%), Gaps = 0/227 (0%)
ecf	Escherichia coli O157:H7 EC4115 (EHEC)	Identities = 206/227 (91%), Positives = 214/227 (94%), Gaps = 0/227 (0%)
ecs	Escherichia coli O157:H7 Sakai (EHEC)	Identities = 206/227 (91%), Positives = 214/227 (94%), Gaps = 0/227 (0%)
ece	Escherichia coli O157:H7 EDL933 (EHEC)	Identities = 206/227 (91%), Positives = 214/227 (94%), Gaps = 0/227 (0%)
elo	Escherichia coli O44:H18 042 (EAEC)	Identities = 206/227 (91%), Positives = 215/227 (95%), Gaps = 0/227 (0%)
eun	Escherichia coli UMNK88	Identities = 205/227 (90%), Positives = 213/227 (94%), Gaps = 0/227 (0%)
setu	Salmonella enterica subsp. enterica serovar Typhimurium U288	Identities = 205/227 (90%), Positives = 214/227 (94%), Gaps = 0/227 (0%)
elr	Escherichia coli O55:H7 RM12579 (EPEC)	Identities = 203/227 (89%), Positives = 212/227 (93%), Gaps = 0/227 (0%)
eok	Escherichia coli O55:H7 CB9615 (EPEC)	Identities = 203/227 (89%), Positives = 212/227 (93%), Gaps = 0/227 (0%)
eoj	Escherichia coli O26:H11 11368 (EHEC)	Identities = 203/227 (89%), Positives = 213/227 (94%), Gaps = 0/227 (0%)
seeb	Salmonella enterica subsp. enterica serovar Bareilly	Identities = 195/227 (86%), Positives = 209/227 (92%), Gaps = 2/227 (1%)
send	Salmonella enterica subsp. enterica serovar Typhimurium DT104	Identities = 190/227 (84%), Positives = 210/227 (93%), Gaps = 1/227 (0%)
setu	Salmonella enterica subsp. enterica serovar Typhimurium U288	Identities = 190/227 (84%), Positives = 210/227 (93%), Gaps = 1/227 (0%)

Supplementary Table 7. List of M.EcoGIX homologs

Summary of BLAST against M.EcoGIX was shown (cut-off at E-value <1.00E-50). BLAST program was run at Kyoto Encyclopedia of Genes and Genom
M.EcoGIX homologs encoded on plasmid are shown in black and those encoded on chromosome are shown in red.

Org code*	Organism	bits	E value	coding replicon	coding region
sef	Salmonella enterica subsp. enterica serovar Typhimurium 798	410	3.00E-143	p798_93	49384..50064
seb	Salmonella enterica subsp. enterica serovar Typhimurium ST4/74	410	3.00E-143	TY474p1	complement(43674..44354)
sej	Salmonella enterica subsp. enterica serovar Typhimurium UK-1	410	3.00E-143	pSTUK-100	31259..31939
sem	Salmonella enterica subsp. enterica serovar Typhimurium T000240	410	3.00E-143	pSTMDT12_L	62102..62782
sey	Salmonella enterica subsp. enterica serovar Typhimurium SL1344	410	3.00E-143	pSLT_SL1344	complement(43674..44354)
seo	Salmonella enterica subsp. enterica serovar Typhimurium 14028S	410	3.00E-143	unnamed	49478..50158
stm	Salmonella enterica subsp. enterica serovar Typhimurium LT2	410	3.00E-143	pSLT	49486..50166
sbz	Salmonella bongori N268-08	410	4.00E-143	RM1	complement(49063..49743)
yph	Yersinia pestis biovar Medievalis Harbin 35	409	1.00E-142	pMT	complement(80298..80975)
ypx	Yersinia pestis D182038	409	1.00E-142	pMT1	89918..90595
ypd	Yersinia pestis D106004	409	1.00E-142	pMT1	88121..88798
ypt	Yersinia pestis A1122	409	1.00E-142	unnamed	complement(41449..42126)
ypz	Yersinia pestis Z176003	409	1.00E-142	pMT1	88123..88800
ypp	Yersinia pestis Pestoides F	409	1.00E-142	MT	130882..131559

Supplementary Table 7. List of M.EcoGIX homologs

Summary of BLAST against M.EcoGIX was shown (cut-off at E-value <1.00E-50).es (KEGG) and their organism code*, along
M.EcoGIX homologs encoded on plasmid are shown in black and those encode

Org code*	Organism	homology
sef	Salmonella enterica subsp. enterica serovar Typhimurium 798	Identities = 190/227 (84%), Positives = 210/227 (93%), Gaps = 1/227 (0%)
seb	Salmonella enterica subsp. enterica serovar Typhimurium ST4/74	Identities = 190/227 (84%), Positives = 210/227 (93%), Gaps = 1/227 (0%)
sej	Salmonella enterica subsp. enterica serovar Typhimurium UK-1	Identities = 190/227 (84%), Positives = 210/227 (93%), Gaps = 1/227 (0%)
sem	Salmonella enterica subsp. enterica serovar Typhimurium T000240	Identities = 190/227 (84%), Positives = 210/227 (93%), Gaps = 1/227 (0%)
sey	Salmonella enterica subsp. enterica serovar Typhimurium SL1344	Identities = 190/227 (84%), Positives = 210/227 (93%), Gaps = 1/227 (0%)
seo	Salmonella enterica subsp. enterica serovar Typhimurium 14028S	Identities = 190/227 (84%), Positives = 210/227 (93%), Gaps = 1/227 (0%)
stm	Salmonella enterica subsp. enterica serovar Typhimurium LT2	Identities = 190/227 (84%), Positives = 210/227 (93%), Gaps = 1/227 (0%)
sbz	Salmonella bongori N268-08	Identities = 191/227 (84%), Positives = 211/227 (93%), Gaps = 1/227 (0%)
yph	Yersinia pestis biovar Medievalis Harbin 35	Identities = 193/227 (85%), Positives = 208/227 (92%), Gaps = 2/227 (1%)
ypx	Yersinia pestis D182038	Identities = 193/227 (85%), Positives =
ypd	Yersinia pestis D106004	Identities = 193/227 (85%), Positives = 208/227 (92%), Gaps = 2/227 (1%)
ypt	Yersinia pestis A1122	Identities = 193/227 (85%), Positives = 208/227 (92%), Gaps = 2/227 (1%)
ypz	Yersinia pestis Z176003	Identities = 193/227 (85%), Positives = 208/227 (92%), Gaps = 2/227 (1%)
ypp	Yersinia pestis Pestoides F	Identities = 193/227 (85%), Positives = 208/227 (92%), Gaps = 2/227 (1%)

Supplementary Table 7. List of M.EcoGIX homologs

Summary of BLAST against M.EcoGIX was shown (cut-off at E-value <1.00E-50). BLAST program was run at Kyoto Encyclopedia of Genes and Genom
M.EcoGIX homologs encoded on plasmid are shown in black and those encoded on chromosome are shown in red.

Org code*	Organism	bits	E value	coding replicon	coding region
ypm	Yersinia pestis 91001 (biovar Microtus)	409	1.00E-142	pMT1	complement(89397..90074)
ypn	Yersinia pestis Nepal516 (biovar Antiqua)	409	1.00E-142	pMT	94790..95467
ypa	Yersinia pestis Antiqua (biovar Antiqua)	409	1.00E-142	pMT	90345..91022
ypk	Yersinia pestis KIM10+ (biovar Mediaevalis)	409	1.00E-142	pMT-1	66648..67325
ype	Yersinia pestis CO92 (biovar Orientalis)	409	1.00E-142	pMT1	complement(64672..65349)
ypg	Yersinia pestis Angola	408	2.00E-142	pMT-pPCP	108442..109119
eun	Escherichia coli UMNK88	403	1.00E-140	pUMNK88_Ent	complement(71868..72521)
ebi	Erwinia billingiae	391	1.00E-135	pEB170	122412..123092
eay	Erwinia amylovora ATCC 49946	388	1.00E-134	p2	13056..13736
eclo	Enterobacter cloacae subsp. cloacae NCTC 9394	340	7.00E-116	chromosome	3619987..3620679
enc	Enterobacter cloacae subsp. cloacae ATCC 13047	339	2.00E-115	pECL_A	53792..54484
kpm	Klebsiella pneumoniae subsp. pneumoniae HS11286	335	7.00E-114	pKPHS2	complement(100181..100882)
kpn	Klebsiella pneumoniae subsp. pneumoniae MGH 78578	333	5.00E-113	pKPN4	52613..53314

Supplementary Table 7. List of M.EcoGIX homologs

Summary of BLAST against M.EcoGIX was shown (cut-off at E-value <1.00E-50).es (KEGG) and their organism code*, along
M.EcoGIX homologs encoded on plasmid are shown in black and those encode

Org code*	Organism	homology
ypm	Yersinia pestis 91001 (biovar Microtus)	Identities = 193/227 (85%), Positives = 208/227 (92%), Gaps = 2/227 (1%)
ypn	Yersinia pestis Nepal516 (biovar Antiqua)	Identities = 193/227 (85%), Positives = 208/227 (92%), Gaps = 2/227 (1%)
ypa	Yersinia pestis Antiqua (biovar Antiqua)	Identities = 193/227 (85%), Positives = 208/227 (92%), Gaps = 2/227 (1%)
ypk	Yersinia pestis KIM10+ (biovar Mediaevalis)	Identities = 193/227 (85%), Positives = 208/227 (92%), Gaps = 2/227 (1%)
ype	Yersinia pestis CO92 (biovar Orientalis)	Identities = 193/227 (85%), Positives = 208/227 (92%), Gaps = 2/227 (1%)
ypg	Yersinia pestis Angola	Identities = 192/227 (85%), Positives = 208/227 (92%), Gaps = 2/227 (1%)
eun	Escherichia coli UMNK88	Identities = 190/203 (94%), Positives = 194/203 (96%), Gaps = 0/203 (0%)
ebi	Erwinia billingiae	Identities = 182/227 (80%), Positives = 200/227 (88%), Gaps = 1/227 (0%)
eay	Erwinia amylovora ATCC 49946	Identities = 180/227 (79%), Positives = 202/227 (89%), Gaps = 1/227 (0%)
eclo	Enterobacter cloacae subsp. cloacae NCTC 9394	Identities = 155/225 (69%), Positives = 184/225 (82%), Gaps = 0/225 (0%)
enc	Enterobacter cloacae subsp. cloacae ATCC 13047	Identities = 155/225 (69%), Positives = 184/225 (82%), Gaps = 0/225 (0%)
kpm	Klebsiella pneumoniae subsp. pneumoniae HS11286	Identities = 164/234 (70%), Positives = 186/234 (79%), Gaps = 8/234 (3%)
kpn	Klebsiella pneumoniae subsp. pneumoniae MGH 78578	Identities = 163/234 (70%), Positives = 185/234 (79%), Gaps = 8/234 (3%)

Supplementary Table 7. List of M.EcoGIX homologs

Summary of BLAST against M.EcoGIX was shown (cut-off at E-value <1.00E-50). BLAST program was run at Kyoto Encyclopedia of Genes and Genom[illegible]
M.EcoGIX homologs encoded on plasmid are shown in black and those encoded on chromosome are shown in red.

Org code*	Organism	bits	E value	coding replicon	coding region
kpa	Klebsiella pneumoniae 30660/NJST258_1	334	5.00E-113	pNJST258N2	10504..11280
kpn	Klebsiella pneumoniae subsp. pneumoniae MGH 78578	331	4.00E-112	pKPN5	complement(9138..9839)
kps	Klebsiella pneumoniae 30684/NJST258_2	320	2.00E-107	pNJST258C1	12170..12946
asa	Aeromonas salmonicida	317	7.00E-107	p5	complement(130148..130792)
kpr	Klebsiella pneumoniae	315	5.00E-106	pKRH	complement(99752..100453)
enr	Enterobacter sp. R4-368	315	6.00E-106	pENT01	complement(110872..111525)
pao	Pantoea sp. At-9b	315	6.00E-106	pPAT9B05	49614..50258
rah	Rahnella sp. Y9602	314	8.00E-106	pRAHAQ02	complement(16681..17325)
srl	Serratia plymuthica 4Rx13	303	2.00E-101	p75	5666..6310
koe	Klebsiella oxytoca E718	296	2.00E-99	pKOX_NDM1	complement(107602..108099)
ecoi	Escherichia coli PMV-1	288	3.00E-96	pHUSEC411like	11492..11995
pva	Pantoea vagans	295	2.00E-90	pPag2	complement(113008..115737)
bgl	Burkholderia glumae	272	4.00E-89	bglu_3p	complement(70623..71276)

Supplementary Table 7. List of M.EcoGIX homologs

Summary of BLAST against M.EcoGIX was shown (cut-off at E-value <1.00E-50).es (KEGG) and their organism code*, along
M.EcoGIX homologs encoded on plasmid are shown in black and those encode

Org code*	Organism	homology
kpa	Klebsiella pneumoniae 30660/NJST258_1	Identities = 163/234 (70%), Positives = 185/234 (79%), Gaps = 8/234 (3%)
kpn	Klebsiella pneumoniae subsp. pneumoniae MGH 78578	Identities = 162/234 (69%), Positives = 184/234 (79%), Gaps = 8/234 (3%)
kps	Klebsiella pneumoniae 30684/NJST258_2	Identities = 161/234 (69%), Positives = 185/234 (79%), Gaps = 8/234 (3%)
asa	Aeromonas salmonicida	Identities = 145/205 (71%), Positives = 169/205 (82%), Gaps = 0/205 (0%)
kpr	Klebsiella pneumoniae	Identities = 150/208 (72%), Positives = 169/208 (81%), Gaps = 7/208 (3%)
enr	Enterobacter sp. R4-368	Identities = 147/217 (68%), Positives = 174/217 (80%), Gaps = 1/217 (0%)
pao	Pantoea sp. At-9b	Identities = 148/211 (70%), Positives = 172/211 (82%), Gaps = 1/211 (0%)
rah	Rahnella sp. Y9602	Identities = 144/211 (68%), Positives = 169/211 (80%), Gaps = 0/211 (0%)
srl	Serratia plymuthica 4Rx13	Identities = 140/201 (70%), Positives = 164/201 (82%), Gaps = 0/201 (0%)
koe	Klebsiella oxytoca E718	Identities = 143/167 (86%), Positives = 153/167 (92%), Gaps = 2/167 (1%)
ecoi	Escherichia coli PMV-1	Identities = 143/170 (84%), Positives = 149/170 (88%), Gaps = 3/170 (2%)
pva	Pantoea vagans	Identities = 133/205 (65%), Positives = 161/205 (79%), Gaps = 6/205 (3%)
bgl	Burkholderia glumae	Identities = 129/217 (59%), Positives = 157/217 (72%), Gaps = 0/217 (0%)

Supplementary Table 7. List of M.EcoGIX homologs

Summary of BLAST against M.EcoGIX was shown (cut-off at E-value <1.00E-50). BLAST program was run at Kyoto Encyclopedia of Genes and Genom
M.EcoGIX homologs encoded on plasmid are shown in black and those encoded on chromosome are shown in red.

Org code*	Organism	bits	E value	coding replicon	coding region
bgd	Burkholderia gladioli	270	3.00E-88	bgla_2p	complement(71826..72482)
bgd	Burkholderia gladioli	268	9.00E-88	bgla_1p	complement(50215..50871)
put	Pusillimonas sp. T7-7	267	1.00E-87	chromosome	271186..271749
bgl	Burkholderia glumae	263	1.00E-85	bglu_4p	complement(73168..73821)
brh	Burkholderia rhizoxinica	263	2.00E-85	pBRH02	44352..45065
nha	Nitrobacter hamburgensis	250	1.00E-80	chromosome	630346..630993
bcj	Burkholderia cenocepacia J2315	249	3.00E-80	pBCJ2315	complement(73946..74599)
bch	Burkholderia cenocepacia HI2424	247	7.00E-78	p1	147027..148010
ecw	Escherichia coli E24377A (ETEC)	241	3.00E-76	pETEC_74	27397..28182
ret	Rhizobium etli CFN 42	236	3.00E-75	chromosome	2062703..2063347
pde	Paracoccus denitrificans	232	2.00E-73	chromosome 2	511449..512126
pde	Paracoccus denitrificans	231	9.00E-73	chromosome 2	338133..338810
pde	Paracoccus denitrificans	228	6.00E-72	chromosome 1	110943..111620

Supplementary Table 7. List of M.EcoGIX homologs

Summary of BLAST against M.EcoGIX was shown (cut-off at E-value <1.00E-50).es (KEGG) and their organism code*, along
M.EcoGIX homologs encoded on plasmid are shown in black and those encode

Org code*	Organism	homology
bgd	Burkholderia gladioli	Identities = 124/207 (60%), Positives = 156/207 (75%), Gaps = 0/207 (0%)
bgd	Burkholderia gladioli	Identities = 123/206 (60%), Positives = 155/206 (75%), Gaps = 0/206 (0%)
put	Pusillimonas sp. T7-7	Identities = 125/187 (67%), Positives = 145/187 (78%), Gaps = 0/187 (0%)
bgl	Burkholderia glumae	Identities = 123/204 (60%), Positives = 150/204 (74%), Gaps = 0/204 (0%)
brh	Burkholderia rhizoxinica	Identities = 124/210 (59%), Positives = 156/210 (74%), Gaps = 4/210 (2%)
nha	Nitrobacter hamburgensis	Identities = 117/201 (58%), Positives = 147/201 (73%), Gaps = 0/201 (0%)
bcj	Burkholderia cenocepacia J2315	Identities = 123/205 (60%), Positives = 152/205 (74%), Gaps = 0/205 (0%)
bch	Burkholderia cenocepacia HI2424	Identities = 121/199 (61%), Positives = 150/199 (75%), Gaps = 0/199 (0%)
ecw	Escherichia coli E24377A (ETEC)	Identities = 108/116 (93%), Positives = 112/116 (97%), Gaps = 0/116 (0%)
ret	Rhizobium etli CFN 42	Identities = 108/202 (53%), Positives = 142/202 (70%), Gaps = 0/202 (0%)
pde	Paracoccus denitrificans	Identities = 107/211 (51%), Positives = 148/211 (70%), Gaps = 0/211 (0%)
pde	Paracoccus denitrificans	Identities = 106/210 (50%), Positives = 146/210 (70%), Gaps = 0/210 (0%)
pde	Paracoccus denitrificans	Identities = 106/210 (50%), Positives = 145/210 (69%), Gaps = 0/210 (0%)

Supplementary Table 7. List of M.EcoGIX homologs

Summary of BLAST against M.EcoGIX was shown (cut-off at E-value <1.00E-50). BLAST program was run at Kyoto Encyclopedia of Genes and Genom[illegible]

M.EcoGIX homologs encoded on plasmid are shown in black and those encoded on chromosome are shown in red.

Org code*	Organism	bits	E value	coding replicon	coding region
gdj	Gluconacetobacter diazotrophicus PAl 5 (JGI)	228	9.00E-72	chromosome	complement(1788916..17895[illegible]
yel	Yersinia enterocolitica LC20	224	2.00E-71	plasmid1_80K	complement(69921..70331)
gdj	Gluconacetobacter diazotrophicus PAl 5 (JGI)	226	6.00E-71	chromosome	complement(1108484..11091[illegible]
gxl	Gluconacetobacter xylinus E25	222	2.00E-69	chromosome	complement(2630354..26310[illegible]
gdi	Gluconacetobacter diazotrophicus PAl 5 (Brazil)	221	7.00E-69	chromosome	393090..393770
goh	Gluconobacter oxydans H24	220	1.00E-68	chromosome	1775734..1776414
gdj	Gluconacetobacter diazotrophicus PAl 5 (JGI)	212	2.00E-65	chromosome	complement(1202456..12031[illegible]
rli	Roseobacter litoralis	202	1.00E-61	chromosome	1945360..1946040
rli	Roseobacter litoralis	195	8.00E-59	chromosome	3396622..3397302
psts	Plautia stali symbiont	173	6.00E-52	pPstS2	8150..8455

Supplementary Table 7. List of M.EcoGIX homologs

Summary of BLAST against M.EcoGIX was shown (cut-off at E-value <1.00E-50).es (KEGG) and their organism code*, along
M.EcoGIX homologs encoded on plasmid are shown in black and those encode

Org code*	Organism	homology
gdj	Gluconacetobacter diazotrophicus PAl 5 (JGI)	Identities = 104/200 (52%), Positives = 140/200 (70%), Gaps = 0/200 (0%)
yel	Yersinia enterocolitica LC20	Identities = 111/135 (82%), Positives = 117/135 (87%), Gaps = 1/135 (1%)
gdj	Gluconacetobacter diazotrophicus PAl 5 (JGI)	Identities = 106/200 (53%), Positives = 140/200 (70%), Gaps = 0/200 (0%)
gxl	Gluconacetobacter xylinus E25	Identities = 104/200 (52%), Positives = 138/200 (69%), Gaps = 0/200 (0%)
gdi	Gluconacetobacter diazotrophicus PAl 5 (Brazil)	Identities = 105/200 (53%), Positives = 138/200 (69%), Gaps = 0/200 (0%)
goh	Gluconobacter oxydans H24	Identities = 102/199 (51%), Positives = 137/199 (69%), Gaps = 0/199 (0%)
gdj	Gluconacetobacter diazotrophicus PAl 5 (JGI)	Identities = 98/200 (49%), Positives = 134/200 (67%), Gaps = 0/200 (0%)
rli	Roseobacter litoralis	Identities = 91/218 (42%), Positives = 136/218 (62%), Gaps = 0/218 (0%)
rli	Roseobacter litoralis	Identities = 86/209 (41%), Positives = 130/209 (62%), Gaps = 0/209 (0%)
psts	Plautia stali symbiont	Identities = 81/101 (80%), Positives = 85/101 (84%), Gaps = 0/101 (0%)
